# Molecular correlates of response to eribulin and pembrolizumab in hormone receptor-positive metastatic breast cancer

**DOI:** 10.1038/s41467-021-25769-z

**Published:** 2021-09-21

**Authors:** Tanya E. Keenan, Jennifer L. Guerriero, Romualdo Barroso-Sousa, Tianyu Li, Tess O’Meara, Anita Giobbie-Hurder, Nabihah Tayob, Jiani Hu, Mariano Severgnini, Judith Agudo, Ines Vaz-Luis, Leilani Anderson, Victoria Attaya, Jihye Park, Jake Conway, Meng Xiao He, Brendan Reardon, Erin Shannon, Gerburg Wulf, Laura M. Spring, Rinath Jeselsohn, Ian Krop, Nancy U. Lin, Ann Partridge, Eric P. Winer, Elizabeth A. Mittendorf, David Liu, Eliezer M. Van Allen, Sara M. Tolaney

**Affiliations:** 1 Department of Medical Oncology, Dana-Farber Cancer Institute, Boston, MA USA; 2 Broad Institute of Massachusetts Institute of Technology and Harvard, Cambridge, MA USA; 3 Breast Oncology Program, Dana-Farber/Brigham and Women’s Cancer Center, Boston, MA USA; 4 Breast Tumor Immunology Laboratory, Department of Cancer Biology, Dana-Farber Cancer Institute, Boston, MA USA; 5 Division of Breast Surgery, Department of Surgery, Brigham and Women’s Hospital, Boston, MA USA; 6 Ludwig Center for Cancer Research at Harvard, Harvard Medical School, Boston, MA USA; 7 Oncology Center, Hospital Sírio-Libanês, Brasília, Brazil; 8 Division of Biostatistics, Department of Data Sciences, Dana-Farber Cancer Institute, Boston, Massachusetts, USA; 9 Center for Immuno-Oncology, Dana-Farber Cancer Institute, Boston, MA USA; 10 Department of Cancer Immunology and Virology, Dana-Farber Cancer Institute, Boston, MA USA; 11 Medical Oncology Department, INSERM Unit 981, Molecular Predictors and New Targets in Oncology, Institut Gustave Roussy, Villejuif, France; 12 Harvard Graduate Program in Biophysics, Boston, MA USA; 13 Hematology/Oncology, Beth Israel Deaconess Medical Center, Boston, MA USA; 14 Breast Cancer, Cancer Center, Massachusetts General Hospital, Boston, MA USA

**Keywords:** Breast cancer, Breast cancer

## Abstract

Immune checkpoint inhibitors (ICIs) have minimal therapeutic effect in hormone receptor-positive (HR+ ) breast cancer. We present final overall survival (OS) results (*n* = 88) from a randomized phase 2 trial of eribulin ± pembrolizumab for patients with metastatic HR+ breast cancer, computationally dissect genomic and/or transcriptomic data from pre-treatment tumors (*n* = 52) for molecular associations with efficacy, and identify cytokine changes differentiating response and ICI-related toxicity (*n* = 58). Despite no improvement in OS with combination therapy (hazard ratio 0.95, 95% CI 0.59–1.55, *p* = 0.84), immune infiltration and antigen presentation distinguished responding tumors, while tumor heterogeneity and estrogen signaling independently associated with resistance. Moreover, patients with ICI-related toxicity had lower levels of immunoregulatory cytokines. Broadly, we establish a framework for ICI response in HR+ breast cancer that warrants diagnostic and therapeutic validation. ClinicalTrials.gov Registration: NCT03051659.

## Abstract (web summary)

A randomized phase 2 clinical trial has recently shown no benefit of the combination eribulin and pembrolizumab over pembrolizumab alone in HR + metastatic breast cancer patients (NCT03051659). Here, the authors are reporting the final OS data and biomarker analyses on a subset of samples to analyze molecular correlates

## Introduction

Patients with hormone receptor-positive (HR+ ) breast cancer resistant to hormonal and targeted therapies represent a large unmet clinical need^1,2^, given the lack effective treatments that extend survival beyond short-lived responses to successive lines of chemotherapy^3^. Immune checkpoint inhibitors (ICIs) have led to significant survival improvements in other solid tumors, including triple-negative breast cancer (TNBC), but have minimal therapeutic effect in HR+ disease^4,5^, even when combined with chemotherapy^6^. The biology underlying ICI resistance in HR+ breast cancer remains incompletely understood.

Intrinsic ICI resistance in HR+ disease is often attributed to the low incidence of molecular features implicated in selective ICI response in other solid tumors^7^, such as PD-L1 positivity^8,9^, mismatch repair deficiency^10^, high tumor mutation burden^11,12^, and immune infiltration^13,14^. However, these conclusions have largely been drawn from studies characterizing HR+ breast cancer independent of treatment, rather than in relation to clinical response to ICIs. The few studies that have investigated HR+ breast cancer treated with ICIs include minimal to no genomic or transcriptomic profiling^4–6,15^.

Clinically, ICIs are likely to advance as therapeutic options in HR+ disease only in combination with established treatments already used in this disease setting, or other agents that augment immune responses. Although the ICI pembrolizumab combined with chemotherapy improved responses in patients with early-stage HR+ breast cancer^16^, our trial is the first randomized study to investigate the combination of an ICI with chemotherapy in patients with metastatic HR+ breast cancer (NCT03051659)^6^. We previously demonstrated that pembrolizumab added to the microtubule-targeting chemotherapy eribulin did not improve objective response rates (ORR) or progression-free survival (PFS)^6^, and here we present the final overall survival (OS) analysis. To determine molecular correlates of differential efficacy in the subset of patients who derived clinical benefit to combination ICI and chemotherapy, we performed an integrated analysis of multi-modal molecular data from trial patients with available tumor tissue and blood samples.

## Results

### No overall survival benefit

**Fig. 1:**
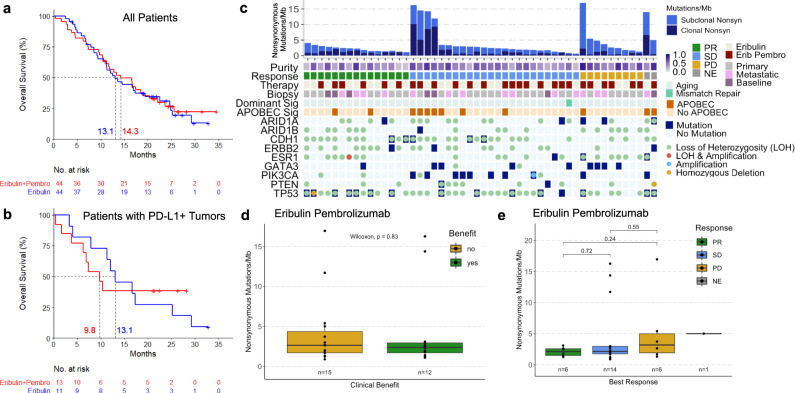
Cohort clinical and genomic characteristics and tumor mutation burden (TMB). **a**, **b** Updated overall survival curves for eribulin ± pembrolizumab in metastatic TNBC show no difference in all patients (**a**) and in patients with PD-L1 + tumors (**b**). **c** Comutation plot shows no association between TMB and response. Each column represents a tumor. Tumors are ordered by RECIST response, and within each response subgroup by decreasing nonsynonymous (Nonsyn) mutational load (top row). Nonsynonymous mutational burden is further subdivided into clonal (dark blue) and subclonal (light blue) mutational load. Tumor purity is the inferred proportion of the tumor sample that is from cancer cells compared to other cell types (Methods). The protocol therapy and biopsy timing (archival primary or metastatic tumor vs. baseline metastatic tumor collected during trial screening) are indicated. Mutational signatures (sig) indicate the dominant signature present at the highest relative proportion and the presence or absence of APOBEC signatures consisting of COSMIC signatures 2 or 13^20,77^. Mutations and copy number alterations in genes commonly mutated in breast cancer are shown for each tumor. **d**, **e** In tumors treated with eribulin and pembrolizumab, nonsynonymous mutational burden was not different in patients with clinical benefit (green) vs. those without clinical benefit (yellow) (**d**) and did not differ by RECIST response (**e**). Unadjusted two-sided Mann–Whitney–Wilcoxon *p*-values are shown. Boxplot limits indicate the interquartile range (IQR; 25th to 75th percentile), with a center line indicating the median. Whiskers show the value ranges up to 1.5 × IQR above the 75th or below the 25th percentile with outliers beyond those ranges shown as individual points. Mb, megabase; NE, not evaluable; PD, progressive disease; PR, partial response; SD, stable disease; TNBC, triple-negative breast cancer.

A total of 88 patients with treatment-refractory, advanced HR+ metastatic breast cancer were randomized 1:1 to eribulin plus pembrolizumab or eribulin monotherapy (Supplementary Fig. 1). All patients except one were female (99%), and the median age (range) was 57 (30–76) years. Of these patients, 61% (54/88) were previously treated with 1-2 chemotherapy regimens for metastatic disease, and 72% (63/88) had liver metastases. With median follow up of 25.8 months (interquartile range [IQR] 21.4–29.8) and deaths in 74% (65/88) of patients, median OS did not differ by treatment arm: 14.3 months (95% confidence interval [CI]: 10.4–19.0) with eribulin plus pembrolizumab vs. 13.1 months (95% CI: 9.4–19.4) with eribulin alone (hazard ratio 0.95, 95% CI: 0.59–1.55, *p* = 0.84; Fig. 1a). A total of 65 patients underwent PD-L1 testing, of which 24 patients (36.9%) had PD-L1+ tumors determined by the modified proportion score (MPS) using the 22C3 antibody. Among these PD-L1+ patients, median OS also did not differ by treatment arm: 9.8 months (95% CI: 3.8–not reached) with eribulin plus pembrolizumab vs. 13.1 months (95% CI: 4.1-25.2) with eribulin (hazard ratio 0.97, 95% CI: 0.37–2.53, *p* = 0.95; Fig. 1b).

### Genomic cohort characteristics

Of the 88 trial patients, 65 had available pretreatment formalin-fixed paraffin-embedded tumor samples for whole-exome sequencing (WES) and whole-transcriptome sequencing (RNA-seq), with matched germline DNA from peripheral blood mononuclear cells. After standard quality control (Methods)^17^, WES data from 50 patients and RNA-seq data from 30 patients were available for analysis (Supplementary Fig. 2a, b). These WES and RNA-seq cohorts had similar clinical outcomes as the overall trial cohort, demonstrated by overlapping confidence intervals (Supplementary Data File 1: Supplementary Table 1). Based on the exploratory nature of these molecular studies, significance tests aside from the gene set enrichment analyses (GSEA) were not corrected for multiple comparisons^18,19^.

Overall, the nonsynonymous tumor mutation burden (TMB) was 2.5 mutations per megabase (Mb), similar to previous reports in breast cancer^11,20,21^. When considering nonsynonymous somatic variants, 30% of tumors had *TP53* mutations, 20% of tumors had *PIK3CA* mutations, and 18% of tumors had *GATA3* mutations (Fig. 1c). The median tumor purity (the proportion of sample DNA from tumor cells) was 0.47 (IQR 0.33–0.61), and the median tumor heterogeneity (the proportion of subclonal mutations) was 0.58 (IQR 0.40–0.81). The median purity-corrected tumor ploidy (the number of chromosome pairs) was 2.10 (IQR 1.97–3.29). Individual tumor characteristics are detailed in Supplementary Data File 1 (Supplementary Table 2).

The predominant mutational signature^22^ in most tumors was related to aging (98% related to aging, 2% related to mismatch repair; Fig. 1c). All 6 tumors with TMB > 10 mutations per Mb had mutational signatures related to the APOBEC family of RNA and DNA-editing enzymes (Fig. 1c), as previously reported^11^. However, the presence of APOBEC mutational signatures was not associated with PFS overall (log rank *p* = 0.73) or in the ICI-treated arm (log rank *p* = 0.23; Supplementary Fig. 3a, b). In the RNA-seq cohort, 13% (4/30) of patients had luminal A tumors as measured by PAM50^23^, 33% (10/30) had luminal B tumors, 30% (9/30) had HER2-enriched tumors, 13% (4/30) had basal tumors, and 10% (3/30) were classified as normal-like tumors with gene expression resembling normal tissue. Individual PAM50 subtypes did not have longer PFS or OS when compared overall (Supplementary Fig. 3c, d), although basal tumors had a higher ORR of 75% (3/4) compared to the ORR of 19% (5/26) in non-basal tumors (Fisher’s exact *p* = 0.048).

To discover genomic features associated with response, we compared patients with clinical benefit (CB, *n* = 24) to those with no clinical benefit (NCB, *n* = 26) overall. CB was defined as complete or partial response by RECIST 1.1^24^ or stable disease ≥6 months, and NCB was defined as progressive disease or stable disease <6 months. To identify features associated with selective response to anti-PD-1 therapy combined with chemotherapy, we focused our analyses on patients treated with eribulin and pembrolizumab who experienced CB (*n* = 12) vs. NCB (*n* = 15), using eribulin-only treated patients with CB (*n* = 12) vs. NCB (*n* = 11) as a non-ICI-treated control group.

### Absence of TMB response association

We next investigated whether TMB correlated with anti-PD-1 response in this HR+ metastatic cohort, based on the association of TMB with ICI response in other tumors^25^, including TNBC^12^. CB patients did not have higher TMB than NCB patients in the ICI-treated cohort (Mann–Whitney–Wilcoxon [MWW] *p* = 0.83; Fig. 1d). Stratification of TMB by RECIST response group confirmed the lack of an association between TMB and response in ICI-treated tumors (MWW *p* = 0.24 between partial response [PR] and progressive disease patients [PD]; Fig. 1e). TMB was also not associated with CB or RECIST response in the overall cohort or in the eribulin alone group (Supplementary Fig. 3e–h). As only 15% (4/27) of patients in the ICI-treated group had high TMB > 10 mutations/Mb, these findings underscore both the lack of direct correlation between TMB as a continuous variable and response to ICI plus eribulin in metastatic HR+ breast cancer, as well as the known low prevalence of high TMB in this disease^11,20,21^, which precluded a powered analysis of high TMB with CB in our cohort.

### Tumor heterogeneity and purity associated with resistance

**Fig. 2:**
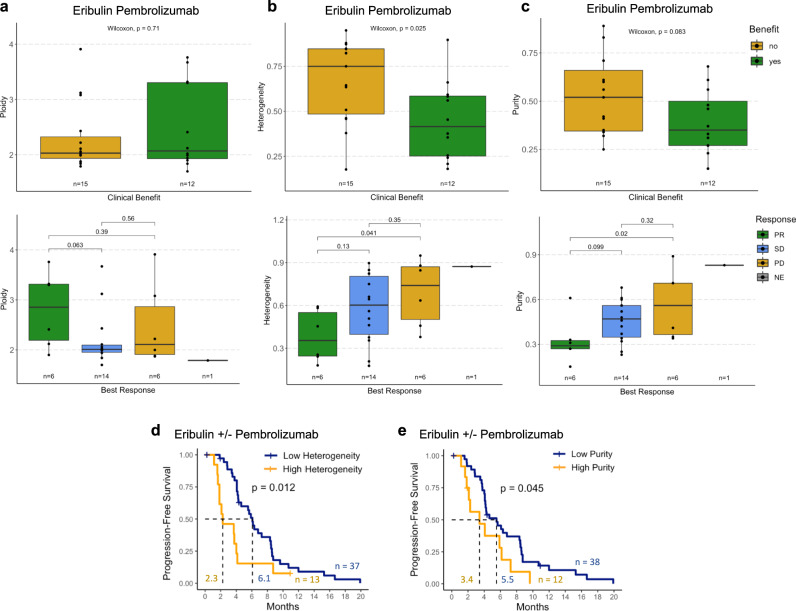
Tumor heterogeneity and purity correlate with resistance to eribulin and pembrolizumab. **a**–**c** Focused analysis of eribulin pembrolizumab WES cohort (*n* = 27): **a** Tumor ploidy, defined as the overall genomic copy number (a normal diploid cell has a copy number of 2; Methods) was not different in patients with clinical benefit (green) versus those without clinical benefit (yellow) or by RECIST response. **b** Tumor heterogeneity, defined as the proportion of subclonal mutations in each tumor (Methods), was lower in patients with clinical benefit (green) versus those without clinical benefit (yellow) and was lower in patients with partial response (PR, green) vs. progressive disease (PD, yellow). **c** Tumor purity, defined as the proportion of DNA from tumor versus other cells in the sample (Methods), was lower in patients with clinical benefit (green) versus those without clinical benefit (yellow) and was lower in patients with partial response (PR, green) vs. progressive disease (PD, yellow). Unadjusted two-sided Mann-Whitney-Wilcoxon p values are shown. Boxplot limits indicate the interquartile range (IQR; 25th to 75th percentile), with a center line indicating the median. Whiskers show the value ranges up to 1.5 × IQR above the 75th or below the 25th percentile with outliers beyond those ranges shown as individual points. **d**, **e** In the overall WES cohort treated with eribulin ± pembrolizumab (*n* = 50), patients with tumors that had top quartile heterogeneity (**d**) and purity (**e**) had longer progression-free survival. Unadjusted two-sided log rank *p*-values are shown. NE, not evaluable; SD, stable disease; WES, whole exome sequencing.

We then examined whether tumor ploidy, heterogeneity, and purity were related to ICI resistance based on prior work showing that these genomic features associated with PD-1 resistance in melanoma^17^. While tumor ploidy was not associated with ICI response (Fig. 2a), nor with overall or eribulin response (Supplementary Fig. 4a), tumor heterogeneity was higher in NCB vs. CB patients (MWW *p* = 0.03) and in PD vs. PR patients (MWW *p* = 0.04) treated with the ICI chemotherapy combination (Fig. 2b). Tumor heterogeneity similarly associated with overall resistance and eribulin resistance (Supplementary Fig. 4b), suggesting that this genomic feature may correlate with general therapeutic resistance rather than ICI-specific resistance. In contrast, tumor purity was selectively associated with ICI resistance in our cohort. Tumor purity trended towards being higher in NCB vs. CB patients (MWW *p* = 0.08) and was higher in PD vs. PR patients (MWW *p* = 0.02) treated with the ICI chemotherapy combination (Fig. 2c). Tumor purity associated with overall resistance but not eribulin resistance (Supplementary Fig. 4c), indicating that the association of tumor purity with resistance may be specific to ICI-based therapies.

In the overall cohort, patients with tumors that had top quartile heterogeneity or purity had shorter PFS (log rank *p* = 0.012 and 0.045, respectively, Fig. 2d, e), even after adjustment for clinical confounders (heterogeneity hazard ratio for progression 3.14, 95% CI: 1.36–7.26, *p* = 0.008; purity hazard ratio 2.25, 95% CI: 0.96–5.24, *p* = 0.06). Within the eribulin pembrolizumab arm, top quartile heterogeneity was not associated with PFS (log rank *p* = 0.1, Supplementary Fig. 4d), perhaps related to the smaller sample size, while patients with tumors that had top quartile purity trended towards having shorter PFS (log rank *p* = 0.054, Supplementary Fig. 4d), even after adjustment for clinical confounders (hazard ratio for progression 4.18, 95% CI: 1.37–12.74, *p* = 0.01).

### Absence of single gene correlates of response

We then performed an unbiased analysis for single-gene predictors of response to ICI-based therapy across all mutated genes detected in this cohort. Before correcting for multiple hypothesis testing, no single-gene nonsynonymous somatic variant was associated with CB to eribulin and pembrolizumab, CB overall, or CB to eribulin alone at unadjusted Fisher’s exact *p* < 0.02 (Supplementary Fig. 5a–c), underscoring the large sample sizes needed for sufficient power to detect these associations^26^. Similarly, no single-gene amplification or homozygous deletion was associated with CB to eribulin and pembrolizumab, CB overall, or CB to eribulin alone at unadjusted Fisher’s exact *p* < 0.04 prior to multiple hypothesis testing correction (Supplementary Fig. 5d–f).

### Immune gene expression associated with response

**Fig. 3:**
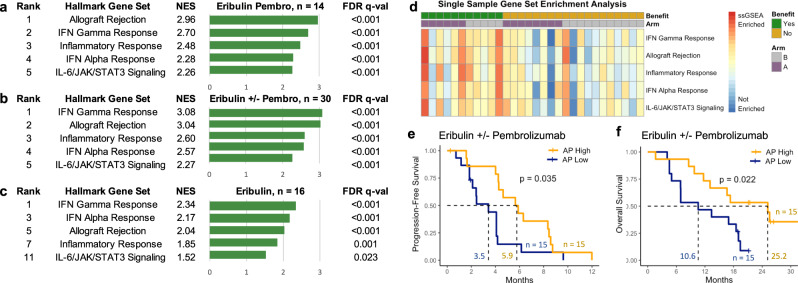
Immune gene set enrichment correlates with response to eribulin and pembrolizumab. **a** For tumors treated with eribulin and pembrolizumab (pembro), the top 5 cancer hallmark gene sets^82^ (GSEA) enriched in patients with clinical benefit (*n* = 6) versus those without clinical benefit (*n* = 8) consisted of immune-related pathways with normalized enrichment scores (NES) > 2.25 and false discovery rate (FDR) *q* values < 0.001. **b** For tumors treated with eribulin ± pembrolizumab, the top 5 cancer hallmark gene sets (GSEA) enriched in patients with clinical benefit (*n* = 11) versus those without clinical benefit (*n* = 19) also consisted of immune-related pathways with NES > 2.25 and FDR *q* values < 0.001. **c** For tumors treated with eribulin alone, the same immune-related gene sets (GSEA) were less enriched in patients with clinical benefit (*n* = 5) versus those without clinical benefit (*n* = 11), as these gene sets were not all ranked in the top 5 enriched gene sets, had lower NES down to 1.52, and larger FDR *q* values up to 0.023. **d** A heatmap of single-sample gene set enrichment analysis (ssGSEA) score, where each column is a tumor arranged first by clinical benefit (green) versus no clinical benefit (yellow) and then by eribulin pembrolizumab treatment arm (purple) versus eribulin treatment arm (gray), shows that tumors with clinical benefit had enrichment of these immune gene sets. Color indicates the ssGSEA score from least enriched (blue) to most enriched (red). **e**, **f** In the overall RNAseq cohort treated with eribulin ± pembrolizumab (*n* = 30), patients with tumors that had high versus low antigen presentation (AP) gene set enrichment scores (ssGSEA; divided by the median) had longer progression-free (**e**) and overall survival (**f**) in unadjusted analyses. All Hallmark pathways^82^ and their GSEA enrichment scores are included in Supplementary Data File 1: Supplementary Table 3, and the antigen presentation gene set is shown in Supplementary Data File 1: Supplementary Table 4. Unadjusted two-sided log rank *p*-values are shown.

In whole transcriptome sequencing data, GSEA using the 50 Hallmark gene sets^27^ revealed that the top five enriched gene sets in tumors from patients with CB vs. NCB to eribulin and pembrolizumab were immune gene sets (false discovery rate (FDR) *q* < 0.001, Supplementary Data File 1: Supplementary Table 3), specifically the allograft rejection, interferon-gamma (IFN-γ), inflammatory response, interferon-alpha (IFN-α), and IL-6/JAK/STAT3 signaling pathways (Fig. 3a). Immune gene sets were also enriched (FDR *q* < 0.001) in patients with CB vs. NCB to eribulin ± pembrolizumab (Fig. 3b) and, to a lesser degree (FDR *q* < 0.001 to <0.05), in patients with CB vs. NCB to eribulin alone (Fig. 3c), consistent with studies indicating that eribulin itself may enhance antitumor immunity^28,29^. Single-sample GSEA (ssGSEA) showed a similar trend towards enrichment of these immune gene sets in CB tumors (Fig. 3d), which had higher allograft rejection and IFN-γ ssGSEA scores (MWW *p* < 0.05, Supplementary Fig. 6a–e), underscoring the link between immune gene expression and response.

Differential expression analysis of the allograft rejection Hallmark gene set, the most enriched gene set in ICI responders, showed that genes with higher expression in CB patients consisted of immune infiltration genes, including the MHC class II genes *HLA-DMA/B* expressed by antigen presenting cells, *CD4* expressed classically by helper T cells, *ITGB2*, an integrin beta chain for T cell adhesion and transmigration, *PTPRC*, which encodes CD45, reflecting greater immune cell infiltration, and *TLR2/3*, encoding toll-like receptors expressed on dendritic cells and other immune cells (MWW *p* < 0.05; Supplementary Fig. 7a). Some of these genes were also more highly expressed in CB patients overall and eribulin-only CB patients (Supplementary Fig. 7b, c).

### Antigen presentation associated with response

In a focused analysis of antigen presentation genes (Supplementary Data File 1: Supplementary Tables 4 and 5), previously found to be associated with ICI response in melanoma^17^, MHC-II-associated HLA genes and *NLRC5*, which positively regulates MHC-I antigen presentation, demonstrated higher expression in tumors with CB vs. NCB to eribulin and pembrolizumab (MWW *p* < 0.05; Supplementary Fig. 7d). Tumors with overall CB and eribulin-specific CB similarly displayed greater expression of MHC-I and II-associated HLA genes (Supplementary Fig. 7e, f). Tumors with overall CB further had elevated antigen presentation ssGSEA scores (MWW *p* = 0.008; Supplementary Fig. 8a). Patients with tumors that had high antigen presentation ssGSEA scores above the median had longer PFS (5.8 vs. 3.4 months, log rank *p* = 0.04, Fig. 3e), even after adjustment for clinical confounders (hazard ratio for progression 0.27, 95% CI: 0.09–0.77, *p* = 0.01), and longer OS (25.2 vs. 10.6 months, log rank *p* = 0.02, Fig. 3f), but not after adjustment for clinical factors (hazard ratio for death 0.40, 95% CI: 0.14–1.15, *p* = 0.09). Above median antigen presentation scores were not associated with PFS in analyses stratified by treatment arm (Supplementary Fig. 8b, c), where small sample sizes limited power.

To determine whether the greater MHC-II gene expression in CB tumors correlated with tumor or immune cells, we evaluated the dendritic cell genes *ITGAX* and *FLT3* and the macrophage gene *ITGAM*, corresponding to the CD11b protein expressed on the surface of macrophages. Of these, only *ITGAX*, encoding the CD11c transmembrane protein found at high levels on dendritic cells, had greater expression in tumors with CB vs. NCB to eribulin and pembrolizumab (transcripts per million [TPM] 138 vs. 63, MWW *p* = 0.02) but not to eribulin alone (median TPM 97 vs. 94, MWW *p* = 0.38, Supplementary Fig. 8d, e). Together with the finding of enriched MHC-II antigen presentation in tumors with CB vs. NCB to eribulin and pembrolizumab, these data suggest that the antigen-presenting role of dendritic cells, which is essential for the priming of T cell responses^30^, may contribute to selective responses to this ICI-chemotherapy regimen.

### Tumor immune infiltration correlates with response

**Fig. 4:**
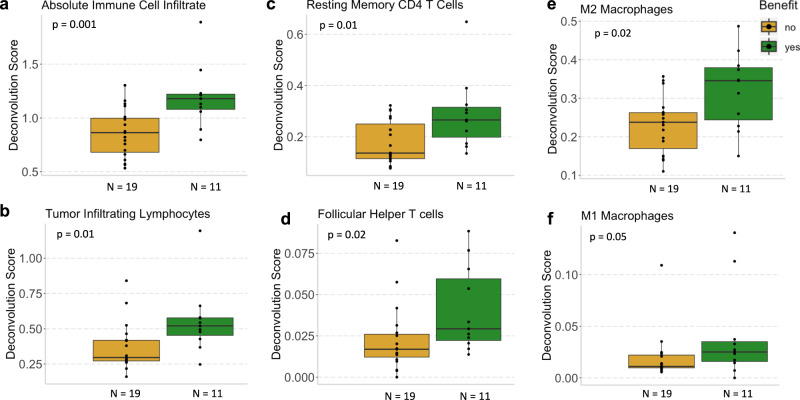
Tumor immune infiltration correlates with response to eribulin ± pembrolizumab. **a** Absolute immune cell infiltrate inferred by CIBERSORTx was higher in patients with clinical benefit (green) versus those without clinical benefit (yellow) to eribulin + /- pembrolizumab. **b** Tumor infiltrating lymphocytes, calculated as the sum of all lymphocytes inferred by CIBERSORTx (Methods), were higher in patients with clinical benefit (green) versus those without clinical benefit (yellow) to eribulin ± pembrolizumab. **c**, **d** Resting memory CD4 + T cells (**c**) and follicular helper T cells (**d**) were higher in patients with clinical benefit (green) versus those without clinical benefit (yellow) to eribulin ± pembrolizumab. **e**, **f** M2 macrophages (**e**) were higher in patients with clinical benefit (green) versus those without clinical benefit (yellow) to eribulin ± pembrolizumab, while M1 macrophages (**f**) trended towards being higher in patients with clinical benefit (green) with borderline significance (*p* = 0.05). Unadjusted two-sided Mann–Whitney–Wilcoxon *p*-values are shown. Boxplot limits indicate the interquartile range (IQR; 25th to 75th percentile), with a center line indicating the median. Whiskers show the value ranges up to 1.5 × IQR above the 75th or below the 25th percentile with outliers beyond those ranges shown as individual points.

Consistent with the immune gene set enrichment in responders, immune cell deconvolution analyses of bulk RNA sequencing data showed larger global immune infiltrates in tumors from CB vs. NCB patients overall (Methods; *p* = 0.001; Fig. 4a) and within each treatment arm (*p* = 0.02 for eribulin pembrolizumab, *p* = 0.03 for eribulin; Supplementary Fig. 9a). The immune cell composition of these infiltrates consisted of both T and myeloid cell populations. Tumor infiltrating lymphocytes were higher in CB vs. NCB tumors overall (*p* = 0.01; Fig. 4b), with a similar trend within each treatment arm (Supplementary Fig. 9b). As for T cell subtypes, CB tumors had more resting memory CD4 T cells (*p* = 0.01, Fig. 4c) and follicular helper T cells (*p* = 0.02; Fig. 4d). With regards to myeloid cell populations, CB tumors had more M2 (pro-tumor) macrophages (*p* = 0.02; Fig. 4e) and trended towards having more M1 (anti-tumor) macrophages (*p* = 0.05; Fig. 4f), implying that the presence of macrophages is associated with CB and that more work is needed to understand the true phenotype of these macrophages.

### Estrogen signaling associated with resistance

**Fig. 5:**
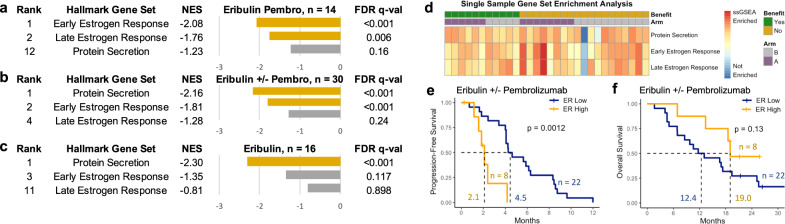
Estrogen signaling correlates with resistance to eribulin and pembrolizumab. **a** For tumors treated with eribulin and pembrolizumab (pembro), the top 2 cancer hallmark gene sets^82^ (GSEA) enriched in patients without clinical benefit (*n* = 8) versus those with clinical benefit (*n* = 6) consisted of estrogen response pathways with normalized enrichment scores (NES) < −1.75 and false discovery rate (FDR) *q* values < 0.01. **b** For tumors treated with eribulin ± pembrolizumab, the top 2 cancer hallmark gene sets (GSEA) enriched in patients without clinical benefit (*n* = 19) versus those with clinical benefit (*n* = 11) consisted of protein secretion and early estrogen response pathways with NES < −1.75 and FDR *q* values < 0.001. **c** For tumors treated with eribulin alone, the top cancer hallmark gene sets (GSEA) enriched in patients without clinical benefit (*n* = 11) versus those with clinical benefit (*n* = 5) was the protein secretion pathway. **d** A heatmap of single-sample GSEA (ssGSEA) score, where each column is a tumor arranged first by clinical benefit (green) versus no clinical benefit (yellow) and then by eribulin pembrolizumab treatment arm (purple) versus eribulin treatment arm (gray), shows that tumors with no clinical benefit had enrichment of these gene sets, particularly estrogen response signaling. Color indicates the ssGSEA score from least enriched (blue) to most enriched (red). **e**, **f** In the overall RNAseq cohort treated with eribulin ± pembrolizumab (*n* = 30), patients with tumors that had top quartile early estrogen response (ER) gene set enrichment scores (ssGSEA) had shorter progression-free (**e**) and overall survival (**f**) in unadjusted analyses. Unadjusted two-sided log rank p values are shown. All Hallmark pathways^82^ and their GSEA enrichment scores are shown in Supplementary Data File 1: Supplementary Table 3.

Transcriptional analysis also indicated molecular determinants of resistance to this ICI-chemotherapy regimen in HR+ breast cancer. Of the Hallmark gene sets^27^, the top two enriched pathways in patients with NCB to eribulin and pembrolizumab were early and late estrogen response pathways (FDR *q* values < 0.001 and 0.006, respectively, Fig. 5a). Early estrogen response gene set enrichment was also associated with NCB to eribulin with or without pembrolizumab overall (FDR *q* value < 0.001, Fig. 5b), while protein secretion gene set enrichment was associated with NCB to eribulin alone (FDR *q* value < 0.001, Fig. 5c).

Additionally, ssGSEA showed a trend towards greater estrogen response gene set enrichment in NCB tumors (Fig. 5d), although this was not statistically significant (Supplementary Fig. 9c, d). Patients with tumors that had top quartile early estrogen response ssGSEA scores had shorter PFS (2.1 vs. 4.5 months, log rank *p* = 0.0012, Fig. 5e), even after adjustment for clinical confounders (hazard ratio for progression 12.2, 95% CI 2.9–52.3, *p* = 0.0007), and a non-significant trend towards better OS (19.0 vs. 12.4 months, log rank *p* = 0.13, Fig. 5f). Furthermore, tumors with top tertile early and late estrogen response ssGSEA scores had lower expression of some antigen presentation genes than tumors with bottom tertile scores (Supplementary Fig. 9e, f; Supplementary Data File 1: Supplementary Table 6), suggesting that reduced antigen presentation may be one immune resistance strategy employed by tumors with high estrogen signaling, as demonstrated by prior preclinical work^31^.

### Integration of genomic and transcriptomic features

**Fig. 6:**
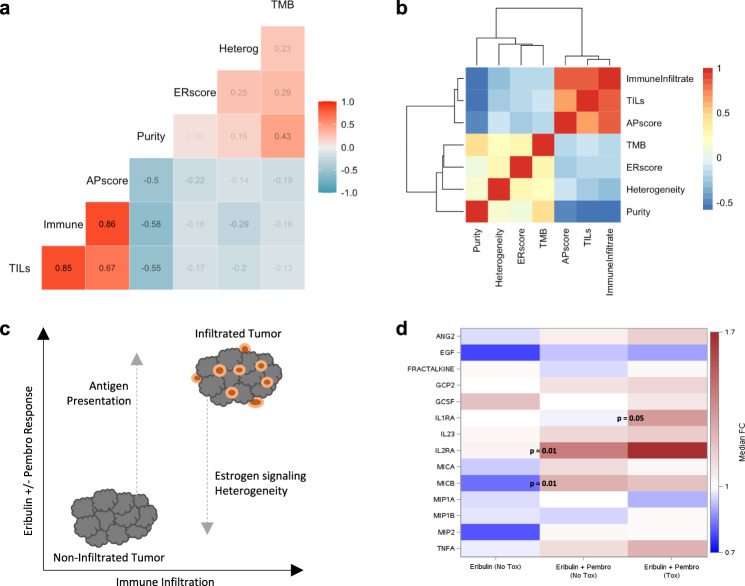
Response correlate interplay and cytokine analyses. **a** Spearman’s correlation coefficients between genomic and transcriptomic features associated with response and **b** hierarchical clustering of these correlation coefficients. Color indicates the Spearman’s correlation between features, from perfect negative correlation (Spearman’s *r* = −1, blue) to perfect positive correlation (Spearman’s *r* = 1, red). Tumor infiltrating lymphocytes (TILs), immune infiltrate, and antigen presentation single-sample gene set enrichment analysis (ssGSEA) score all clustered together, while tumor purity negatively correlated with this cluster. Tumor heterogeneity and estrogen response ssGSEA score were independent from the immune-infiltrated cluster and tumor purity. The sample size for each correlation depended on the number of available data points: correlations involving exclusively genomic or transcriptomic data had *n* = 50 or 30 tumor samples, respectively, whereas correlations involving genomic and transcriptomic features had *n* = 28 tumor samples with data available. **c** Schematic representation of potential interplay of genomic and transcriptomic features. Antigen presentation may increase responses in non-immune infiltrated tumors, while heterogeneity and estrogen signaling may reduce responses in immune-infiltrated tumors. **d** Heatmap of median pre- to on-treatment fold changes in cytokines across three immune-related toxicity groups: patients treated with (1) eribulin or (2) eribulin and pembrolizumab with no immune-related toxicity versus patients treated with (3) eribulin and pembrolizumab with immune-related toxicity. Shown are unadjusted two-sided Mann–Whitney–Wilcoxon *p*-values for significant fold-change (FC) differences between adjacent groups. The sample size for each group depended on the number of available data points and ranged from 15–19 patients as indicated in Supplementary Data File 1: Supplementary Tables 10–11. AP, antigen presentation; ER, estrogen response; TMB, tumor mutational burden; tox, toxicity.

To understand the interplay between the genomic and transcriptomic features we identified, we calculated the Spearman’s correlation coefficients between response predictors (Fig. 6a) and performed hierarchical clustering on these correlation coefficients (Fig. 6b). Tumor infiltrating lymphocytes, immune infiltrate, and antigen presentation score all clustered together, suggesting the same underlying biology. Tumor purity was negatively correlated with this cluster, suggesting that high tumor purity reflects lower immune infiltration, as previously shown in melanoma^17^. Tumor heterogeneity and estrogen response score were independent from the immune-infiltrated cluster and tumor purity, implying distinct biological mechanisms of resistance. Altogether, these results suggest a framework for ICI response in HR+ breast cancer in which greater antigen presentation augments responses in non-immune infiltrated tumors, while genomic heterogeneity and estrogen signaling reduce responses in immune-infiltrated tumors (Fig. 6c). This framework identifies potential ICI combination strategies for enhancing the antitumor immune response in HR+ breast cancer, including partner agents that enhance antigen presentation, for instance by inducing innate immune responses, and those that block estrogen signaling, namely endocrine therapies.

### Immunosuppressive cytokine associated with resistance

Given the role of cytokines in regulating immune responses coupled with previous studies showing a link between circulating cytokines and ICI response in melanoma^32,33^, we hypothesized that immune-related cytokines may correlate with response and analyzed pre- and on-treatment plasma samples in patients with CB vs. NCB, adjusted for treatment arm (Methods). Of 58 patients with pre-treatment cytokine analyses, 38 (66%) were treated with eribulin and pembrolizumab, while 20 (34%) were treated with eribulin alone. Of these patients, 30 (52%) had CB, while 28 (48%) had NCB. Of the 14 cytokines passing quality control and adjusted for batch effect and treatment arm, only the immunosuppressive cytokine MHC class I polypeptide-related sequence B (MICB), which dampens natural killer and T cell activation^34,35^, showed a trend towards higher pre- and on-treatment levels in patients with NCB (Van Elteren *p* = 0.06 and 0.02, respectively; Supplementary Fig. 10a, Supplementary Data File 1: Supplementary Table 7).

### Reduced immunoregulatory cytokines in immune-related toxicity

Based on prior work showing that cytokines may predict immune-related adverse events (irAEs) in other solid tumors^36,37^, we hypothesized that cytokine changes may also distinguish irAEs in breast cancer and analyzed matched pre- and on-treatment plasma samples in pembrolizumab-treated patients with irAEs compared to those without irAEs (Methods). Of the 44 patients in the trial treated with eribulin and pembrolizumab, 21 (48%) had irAEs, including 2 patients who experienced both immune-related colitis and grade 5 sepsis^6^. Compared to other trials of ICIs with chemotherapy in metastatic breast cancer, this irAE frequency was similar to ENHANCE-1 (pembrolizumab with eribulin; 43%)^38^ and IMpassion130 (atezolizumab with nab-paclitaxel; 57%)^8^ but higher than KELLY (pembrolizumab with eribulin; 25%)^39^ and KEYNOTE-355 (pembrolizumab with nab-paclitaxel, paclitaxel, or gemcitabine plus carboplatin; 26%)^40^, indicating wide heterogeneity that may be attributable to the lack of a standardized definition for irAEs^41,42^. In the present trial, patients with vs. without irAEs had no difference in PFS or OS (HR for progression 0.8, 95% CI 0.5–1.4, *p* = 0.5; HR for death 0.8, 95% CI 0.4–1.4, *p* = 0.4; Supplementary Data File 1: Supplementary Table 8), which differs from previous studies showing that irAEs correlate with improved ICI responses^43,44^, likely because our analysis addressed the possibility of guarantee-time bias^45^.

At the pre-treatment baseline timepoint, only the chemokine CCL3 (also known as macrophage inflammatory protein 1-alpha [MIP1a]), which has been associated with irAEs in melanoma^36,46^, had higher levels in patients with vs. without irAEs (MWW *p* = 0.0006, Supplementary Fig. 10b, Supplementary Data File 1: Supplementary Table 9). Conversely, five other immunoregulatory cytokines had lower pre-treatment baseline levels in patients with vs. without irAEs, specifically MICB, interleukin-2 receptor alpha chain (IL-2RA), interleukin-23 (IL-23), angiopoietin-2 (Ang2), and CX3CL1, also known as fractalkine (Supplementary Fig. 10c–g, Supplementary Data File 1: Supplementary Table 9), mirroring prior work showing lower baseline levels of immunoregulatory cytokines in patients with irAEs^37^. These same five cytokines also had lower on-treatment levels in patients with vs. without irAEs at timepoints matched to irAE occurrence (Supplementary Fig. 10c–g, Supplementary Data File 1: Supplementary Table 9), suggesting a possible protective effect against immune-related toxicity. The immunosuppressive cytokine interleukin-1 receptor antagonist (IL-1RA) had a greater fold-change increase in patients with irAEs compared to those without irAEs (33% increase vs. no change, unadjusted MWW *p* = 0.05; Fig. 6d, Supplementary Data File 1: Supplementary Table 10), in line with other work showing melanoma patients who experience irAEs have increased levels of IL-1RA^36,46^.

To contextualize these changes and isolate the effect of pembrolizumab, we measured on-treatment cytokine changes in patients receiving eribulin and pembrolizumab who did not experience immune-related toxicity compared to patients treated with eribulin alone as a control group. Paired analyses of matched pre- and post-treatment samples showed that both IL-2RA and MICB increased in patients on eribulin and pembrolizumab (median fold-change increases of 43% and 27%, Wilcoxon signed-rank *p* = 0.0005 and 0.03 compared to no change, respectively, Supplementary Data File 1: Supplementary Table 11). These two cytokines also had greater on-treatment increases in patients treated with combination therapy compared to patients treated with eribulin alone (MWW *p* = 0.01 for both, Fig. 6d, Supplementary Data File 1: Supplementary Table 11). The increases in IL-2RA and MICB observed with pembrolizumab exposure, in conjunction with their lower baseline and on-treatment levels in patients with vs. without irAEs, indicate that these cytokines may mediate tighter immune regulation in response to pembrolizumab that may contribute to the prevention of immune-related toxicity and, in the case of higher MICB, also worse ICI responses.

## Discussion

In this study, we analyzed multimodal molecular data from patients with metastatic HR+ breast cancer treated with pembrolizumab and eribulin compared to eribulin alone. Although there was no OS difference in the randomized trial (hazard ratio 0.95, 95% CI: 0.59–1.55, *p* = 0.84), the results of this analysis elucidate molecular mechanisms contributing to the critical dilemma of ICI resistance in HR+ disease. Our data show that ICI resistant HR+ tumors not only have lower levels of immune infiltration but also greater tumor heterogeneity and estrogen signaling (Fig. 6c), as well as lower antigen presentation that may be mediated in part by immune cells, specifically fewer dendritic cells. We further demonstrate that patients with irAEs had lower levels of immunosuppressive cytokines, suggesting that looser immune regulation may increase the risk of immune-related toxicity.

Our results highlight several tumor-intrinsic mechanisms of resistance ranging from genomic characteristics to estrogen signaling. Tumor heterogeneity and tumor purity were associated with ICI resistance, as previously found in melanoma^17^. Tumor heterogeneity has been linked to poor prognosis across multiple tumor types and treatments^47–49^, as heterogeneous tumors have more subclonal mutations and higher rates of mutagenesis with increased probability of preexisting or rapidly evolving resistance. As tumor heterogeneity was associated with resistance to both combination therapy and eribulin alone, tumor heterogeneity may be a marker of general therapy resistance rather than ICI-specific resistance. Nonetheless, these results raise the question of whether higher tumor heterogeneity contributes to the lower ICI response rates observed in more heavily treated HR+ breast cancers compared to treatment-naïve tumors^6,16^. In contrast, high tumor purity was associated with resistance to ICI therapy but not with resistance to eribulin alone and therefore may reflect a weaker anti-cancer immune response, as indicated by the inverse correlation between tumor purity and immune infiltration. Moreover, our findings that high estrogen signaling associated with ICI resistance and reduced antigen presentation not only mirror previous work showing that oncogenic signaling induces ICI resistance^50–55^, but also raise a possible mechanism of immune exclusion by which HR+ breast cancer evades T cell recognition by hiding tumor antigens. These data suggest that endocrine therapies to block estrogen signaling may enhance ICI responses in this patient population.

Our transcriptomic data further showed that immune infiltration and antigen presentation associated with response, supporting extensive prior work in other solid tumors^14,56–62^. While certain intratumoral T cell infiltration patterns correlate with unfavorable outcomes in early-stage HR+ breast cancer^63–65^, our finding of immune gene enrichment in tumors with improved responses to both ICI and non-ICI regimens suggests that immune infiltration may delineate metastatic HR+ tumors with a higher probability of general therapeutic response. The finding that M2 (pro-tumor) and M1 (anti-tumor) macrophages both associated with benefit highlights the complexity of tumor associated macrophages, as the M2/M1 dichotomy does not reflect the plasticity of macrophages in the tumor microenvironment and warrants further analysis at the single cell level^66,67^. Further, our observation that antigen presentation correlates with response matches a prior report of HLA class I expression predicting response to chemotherapy in early-stage HR+ disease^68^. The association of antigen presentation with improved survival in our study may be related to the inclusion of HLA class II expression, as indicated by prior studies demonstrating the importance of MHC-II in ICI response in other solid tumors^17,62,69^. The greater *ITGAX* expression in our ICI responders indicates that higher MHC-II gene expression may be mediated by dendritic cells and calls for single cell transcriptomic studies to confirm this finding.

Lastly, our plasma proteomic analysis demonstrated higher levels of several immunoregulatory cytokines in patients without irAEs, suggesting that these cytokines may have mediated a protective effect against immune-related toxicity, such as IL-2RA, in maintaining regulatory T cells^70^, and IL-23, in maintaining intestinal barrier function^71^. Our findings of lower circulating IL-2RA and IL-23 in patients with irAEs is consistent with the reduced *IL2*, *IL23A*, and *IL23R* gene expression found in colon samples from patients with ICI-related colitis^72^. In our patients with irAEs, the greater baseline level of MIP1a and on-treatment increase in IL-1RA also echo prior studies that found amplified levels of these cytokines in patients with irAEs^36,46,73^. Notably, circulating biomarker analyses in melanoma patients demonstrated that elevated fractalkine contributed to a gene signature predictive of irAEs^36^ and that increases in IL-2RA coincided with the development of symptomatic pneumonitis^73^. In contrast, we identified that fractalkine and IL-2RA were higher in metastatic HR+ breast cancer patients who did not experience irAEs, which underscores the importance of investigating disease-specific ICI-related toxicity.

In light of the small sample size and lack of multiple comparisons correction in all but the GSEA findings, our results require validation in independent and larger cohorts, as minimal data were publicly available on molecularly sequenced HR+ tumors treated with ICIs for validation at the time of this study. Further, as mostly archival tumor specimens were available for study, the somatic alteration and transcriptional program landscapes may have further evolved prior to ICI treatment, and these changes would not be captured by the current study. In addition, our findings may not reflect mechanisms underlying response to ICI monotherapy, as the ICI regimen included chemotherapy and the treatment arms showed no difference in clinical outcomes. Finally, while the overall trial was powered to conclude no clinical benefit of adding pembrolizumab to eribulin, the small molecular cohort sizes have limited power to conclude that the negative molecular findings are true negatives rather than false negatives. In fact, the lack of immunotherapy benefit observed in our small PD-L1-positive cohort may be a false negative, based on larger trials in metastatic triple-negative breast cancer showing that immunotherapy added to chemotherapy improves clinical outcomes in patients with PD-L1-positive disease^8,40^. This question of whether immunotherapy benefits PD-L1-positive hormone-receptor positive breast cancer will be better answered by the ongoing randomized trial of the antibody drug conjugate sacituzumab govitecan ± pembrolizumab in 110 patients with PD-L1-positive metastatic hormone receptor-positive breast cancer (NCT04448886).

Our study nonetheless demonstrates the value of integrating rich clinical data with molecular tumor characterization, and it provides critical insights into immunogenomic mechanisms underlying therapeutic response in HR+ breast cancer. If these results are confirmed, tumor heterogeneity and immune infiltration could contribute to a tumor classification system to better select patients for ICI-based therapies, and ICI combination agents that promote antigen presentation or reduce estrogen signaling, such as endocrine therapies, should be explored in clinical trials as partner agents to augment the clinical benefit of ICIs for patients with HR+ tumors (Fig. 6c). This study thus provides a foundation for the development of evidence-based therapeutic selection strategies and rationale combination regimens to combat ICI resistance in HR+ breast cancer.

## Methods

### Patient cohort and clinical endpoints

The overall trial was a randomized, open-label, phase II study of eribulin mesylate with or without pembrolizumab for patients with HR+/HER2− metastatic breast cancer^6^. Eligible patients had received 0–2 prior lines of chemotherapy in the advanced disease setting and were required to have received at least 2 prior lines of endocrine therapy in either the adjuvant or metastatic setting, unless the treating physician determined that they were not appropriate candidates for endocrine therapy.

Patients received eribulin 1.4 mg/m^2^ on days 1 and 8 of each 21-day cycle with or without pembrolizumab 200 mg IV on day 1 of each 21-day cycle. ORR was determined using RECIST v1.1 criteria^24^. PFS was defined as the time between the date of study randomization and the date of documented disease progression or death due to any cause. For patients without documentation of progression or death, PFS was censored on the last date the patient was known to be alive without progression prior to the start of another systemic therapy. OS was defined as the time between the date of protocol therapy initiation and the date of death from any cause. For subjects without documentation of death, OS was censored on the last date the patient was known to be alive.

Participating centers consisted of Dana-Farber Cancer Institute, Massachusetts General Hospital, and Beth-Israel Deaconess Medical Center. The institutional review board (IRB) at each institution approved the study protocol, and all patients provided written informed consent prior to study entry. The study was monitored by the Dana-Farber/Harvard Cancer Center Data Safety Monitoring Board and complied with all ethical regulations.

### Samples

Samples were collected retrospectively and prospectively at the participating sites. Excision or biopsy of breast cancer tissue was preserved as formalin-fixed and paraffin-embedded (FFPE) specimens. Of the 52 samples with either WES or RNA-seq data, 56% (28/52) were archival primary tumors, 30% (15/52) were metastatic tumors, and 14% (7/52) were obtained at baseline prior to initiating protocol therapy.

### PD-L1 testing

Tumors from 65/88 (74%) patients underwent PD-L1 testing assessed centrally by QualTek using the 22C3 antibody. Results were reported as the modified proportion score (MPS), defined as the proportion of cells, including both tumor and mononuclear inflammatory cells, located within tumor nests that stained for PD-L1. A tumor sample was determined to be PD-L1 positive if the MPS score was ≥1. The MPS score was used here and in the original publication of the trial results^6^, because it was the standard PD-L1 assay for pembrolizumab at the time of the trial.

### Whole exome sequencing

Whole exome sequencing was performed at the Broad Institute on formalin-fixed paraffin-embedded tumor samples using Illumina’s ICE hybrid-capture bait set^17,26,74^. Germline DNA was obtained from peripheral blood mononuclear cells. Tumor and germline DNA were sequenced on an Illumina HiSeq 2500 with a 76 base paired-end sequencing protocol, targeting a coverage depth of 50x for tumor samples and 25x for germline samples. Exome sequencing data alignment and initial processing was performed using the Broad Institute Picard pipeline. BAM files were uploaded into Terra (https://app.terra.bio/). Sequencing data were passed through additional quality control and processing methods in Terra. Quality-control (QC) cutoffs were mean target coverage > 45X (tumor) and > 20X (matched normal; GATK Depth of Coverage^75^), cross-contamination of samples estimation (ContEst^76^) < 10%, tumor purity (ABSOLUTE^77^, FACETS^78^) ≥ 10%, and tumor-in-normal contamination (deTIN^79^) < 30%. A total of 3 samples were excluded, because they did not meet these QC criteria (Supplementary Fig. 2a).

We used an adaptation of the Getz Lab Cancer Genome Analysis WES pipeline (https://docs.google.com/document/d/1VO2kX_fgfUd0x3mBS9NjLUWGZu794WbTepBel3cBg08) developed at the Broad Institute to call, filter and annotate somatic mutations with modifications to enhance variant classification. For variant calling, the MuTect 1.0 method^80^ was employed to identify somatic single-nucleotide variants with computational filtering of artifacts introduced by DNA fixation procedures^75^ and DNA oxidation during sequencing^81^. Strelka was used to identify small insertions or deletions^82^, and panel of normal filtering was utilized for rare artifacts specific to the bait set used^80^. Oncotator was applied to annotate identified alterations^83^. All identified mutations are included in the Supplementary Source Data file.

Tumor mutation burden was defined as the nonsynonymous mutational burden normalized by megabases covered at adequate depth to detect variants with 80% power using MuTect given estimated tumor purity by ABSOLUTE^26^. The number of bases covered at a given depth threshold in the tumor was determined using the GATK DepthOfCoverage method^75^. Tumor purity and ploidy were estimated using ABSOLUTE^77^ and FACETS^78^. Tumor heterogeneity was defined as the proportion of mutations in each sample that were inferred to be subclonal. As per our prior work^17^, subclonal mutations were identified as those having a cancer cell fraction < 0.8 estimated by ABSOLUTE^77^. The R package deconstructSigs^84^ was applied to identify mutational signatures of individual tumor samples using the validated COSMIC signatures (mutational signatures version 2) previously found in breast cancer (signatures 1, 2, 3, 5, 6, 8, 10, 13, 17, 18, 20, 26, and 30)^22,85^.

The total number of copy number alterations for each tumor was calculated using an adapted binary segmentation method (ReCapSeg)^86^, and genes were annotated with Oncotator^83^. Allelic copy number alterations were identified by incorporating heterozygous single-nucleotide polymorphisms into the binary segmentation method (Allelic CapSeg). Allelic segments were adjusted for tumor purity and ploidy. Allelic amplifications and deletions were then called integrating the purity- and ploidy-corrected allelic copy number, and gene-level copy number alterations were determined (Supplementary Source Data file)^77^.

### Whole transcriptome sequencing

Whole transcriptome sequencing was performed at the Broad Institute on FFPE samples with sufficient RNA quality (DV200 > 20%) using strand specific transcriptome capture^17,74^. RNA was sequenced on an Illumina HiSeq 2500 with a 101 base paired-end sequencing protocol, targeting a depth of 50 million reads. RNA-seq results were aligned with STAR and then quantified with RSEM to yield gene-level expression in transcripts per million (TPM)^87,88^, included in the Supplementary Source Data file. RNA-seq QC cutoffs included > 10 million absolute number of reads, >15,000 genes detected, and >10% of total reads with ambiguous alignment. A total of 4 samples were excluded, because they did not meet these QC criteria (Supplementary Fig. 2b). The following alignment metrics were also considered: percentage of uniquely mapped reads, average mapped read length, number of splices, mismatch rate per base, percentage of multi-mapped reads, percentage of reads mapped to too many locations, percentage of unmapped reads due to too many mismatches, percentage of unmapped reads due to reads being too short, and percentage of unmapped reads due to other reasons. The remaining samples were clustered across these alignment metrics using principal-component analysis, which revealed no outlier samples.

PAM50 subtype was computed using the R package genefu^89^. Gene pathway expression was evaluated with GSEA^27^ (https://gsea-msigdb.github.io/gsea-gpmodule/v20/index.html) using the Hallmark gene sets from the Molecular Signatures Database^90^ with upper quantile normalized TPM values and 1,000 gene set permutations on the Gene Pattern website (https://cloud.genepattern.org/). Also on the Gene Pattern website, ssGSEA^91^ (https://gsea-msigdb.github.io/ssGSEA-gpmodule/v10/index.html) was performed to generate nonparametric gene set scores for individual samples, included in the Supplementary Source Data file. Tumor immune cell composition was determined with the original CIBERSORT^92^ method within the CIBERSORTx deconvolution algorithm (https://cibersortx.stanford.edu/)^93^, inputting the RNA-seq TPM matrix for the cohort and using absolute mode on the LM22 gene set with quantile normalization disabled, 1,000 permutations, and B mode batch correction to correct for the batch differences between the RNA-seq data in this study and the LM22 signature, which was derived from microarray data. These results are included in the Supplementary Source Data file. Tumor infiltrating lymphocytes were calculated as the sum of T cells, NK cells, B cells, and plasma cells inferred by CIBERSORTx^93^.

### Statistical analysis

Continuous molecular variables were compared between CB vs. NCB groups using the non-parametric MWW test with the *wilcox.test()* or *stat_compare_means(method* = *‘wilcox’)* R function. The proportion of tumors with mutation and copy number alterations were compared with two-sided Fisher’s exact tests (*fisher.test()* R function). Two-sided 95% confidence intervals for ORRs were calculated with the Clopper-Pearson method (*clopper.pearson.ci()* R function). Boxplots and volcano plots were created with the R packages gplots and ggplot2. Kaplan-Meier analyses and Cox proportional-hazards models (*coxph()* R function) were performed with the R packages survival and survminer to evaluate the association of molecular correlates with survival. The median follow up time was estimated using the Kaplan-Meier estimate of the censoring function. The IQR of the follow-up was also estimated from the Kaplan-Meier estimate using the 25th and 75th percentiles of the distribution. Cox proportional-hazards models were adjusted for clinical confounders, specifically age, performance status, prior lines of chemotherapy, and bone-only metastases. Significance testing for differences in PFS and OS were calculated using the log-rank test (*ggsurvplot (survfit())* R function) at a significance level of *p* < 0.05. To address the potential for guarantee-time bias^45^, an extended Cox proportional-hazards analysis with time-dependent covariates and one- and two-month conditional landmark analyses were performed for the calculation of PFS and OS classified by the incidence of irAEs. All comparisons were two-sided with an alpha level of 0.05. The false discovery rate for GSEA was controlled with the Benjamini-Hochberg method using a threshold of *q* < 0.05. Spearman’s correlation coefficients were calculated with the *ggcorr (method* = ‘*spearman’)* R function, and hierarchical clustering was performed using the *heatmap()* R function. All statistical analyses were performed in SAS 9.4 and R 3.5.1.

### Plasma proteomic samples

We investigated changes in cytokines between patients with CB vs. NCB and between ICI-chemotherapy-treated patients with vs. without irAEs compared to chemotherapy-only treated patients. Of the 88 patients enrolled in the trial, 20 patients treated with eribulin and pembrolizumab and 1 patient treated with pembrolizumab alone in cross-over were identified as having experienced grade 2 or above immune-related toxicity, of which 19 had paired baseline and on-treatment at the time of irAE plasma samples. The timepoint closest to the irAE was used as the reading at first AE, unless the baseline timepoint was closest. In that event, the second blood draw timepoint was used, which occurred for 10 patients. Baseline and matching on-treatment plasma samples at the time of first irAE were obtained from 19 patients treated with combination therapy who did not experience irAEs, as well as 19 patients treated with eribulin alone.

#### Analysis

These plasma samples were analyzed using the FLEXMAP 3D Luminex multiplex cytokine analysis platform xPONENT. A dilution factor of 2x was used for every cytokine. Samples that had readings lower than the lowest standards were excluded from the analysis. Data for the eribulin and pembrolizumab group without irAEs was obtained at a later date, so batch correction was performed using an established algorithm to align the measurements from the two batches^94^. This batch corrected cytokine data is available in the Supplementary Source Data file. Comparisons by CB were adjusted for treatment arm with non-parametric Van Elteren tests, and comparisons by irAE groups were calculated with non-parametric MWW tests. All p-values are nominal and have not been adjusted for multiple comparisons.

### Reporting summary

Further information on research design is available in the Nature Research Reporting Summary linked to this article.

## Supplementary information

Supplementary Information

Description of Additional Supplementary Files

Supplementary Data 1

Reporting Summary

## Data Availability

All reasonable requests for raw and analyzed data and materials will be promptly reviewed by the senior authors to determine whether the request is subject to any intellectual property or confidentiality obligations. Patient-related data not included in the paper may be subject to patient confidentiality. Any data and materials that can be shared will be released via a material transfer agreement. Source data are provided with this paper. All analyzed sequencing data are provided in the Supplementary Information. The raw sequencing data in this study are available in the dbGaP database under accession code phs002419.v1.p1. Data dictionaries and variable summaries are available on the dbGaP FTP site: https://ftp.ncbi.nlm.nih.gov/dbgap/studies/phs002419/phs002419.v1.p1. Source data are provided with this paper.

## Source data

Source Data
