# Supplementary material for: Molecular correlates of response to eribulin and pembrolizumab in hormone receptor-positive metastatic breast cancer
Source: Nat Commun. 2021 Sep 21;12:5563. doi: 10.1038/s41467-021-25769-z (PMC8455578; doi:10.1038/s41467-021-25769-z)

Supplementary Figures

Supplementary Fig. 1: Trial Consort Diagram
Supplementary Fig. 2: Genomic Cohort Consort Diagram
Supplementary Fig. 3: Genomic Cohort Survival and Tumor Mutation Burden
Supplementary Fig. 4: Tumor Ploidy, Heterogeneity, and Purity by Benefit and Response
Supplementary Fig. 5: Single-Gene Mutations and Copy Number Alterations
Supplementary Fig. 6: Immune Gene Set Single-Sample Gene Set Enrichment Analysis
Supplementary Fig. 7: Gene Expression Volcano Plots by Benefit
Supplementary Fig. 8: Antigen Presentation Scores and Immune Cell Markers
Supplementary Fig. 9: Immune Infiltration and Estrogen Response Gene Expression
Supplementary Fig. 10: Cytokines by Response and Toxicity

Supplementary Fig. 1: Trial Consort Diagram

Consort diagram for final overall survival analysis of randomized phase II trial of eribulin +/- pembrolizumab in patients with metastatic HR+ HER2- breast cancer (NCT03051659).

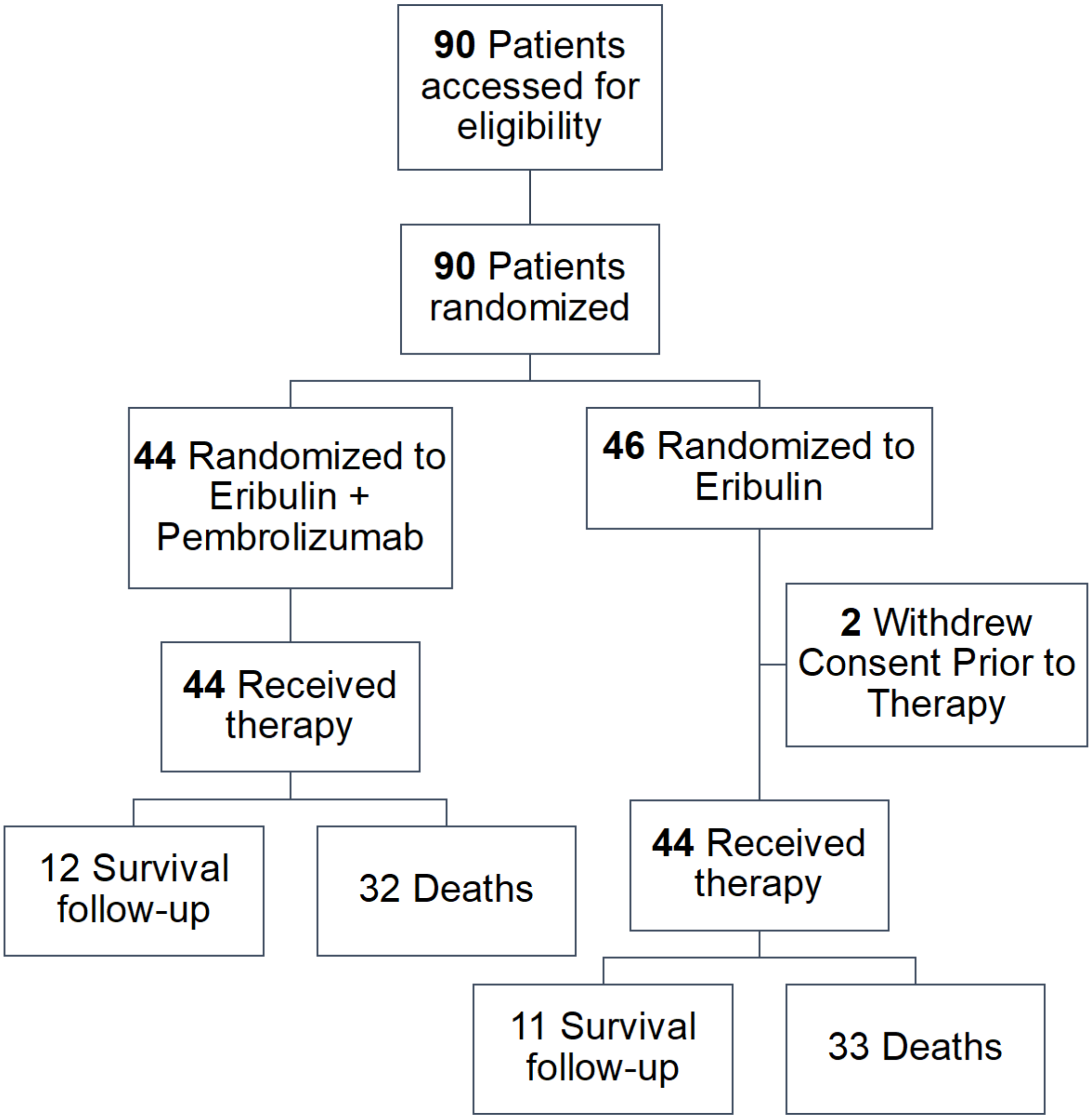

Supplementary Fig. 2: Genomic Cohort Consort Diagram
Sample inclusion and exclusion criteria, including quality control filtering, for tumors included in the (**a**) whole exome sequencing (WES) and (**b**) RNA sequencing (RNA-seq) cohorts.

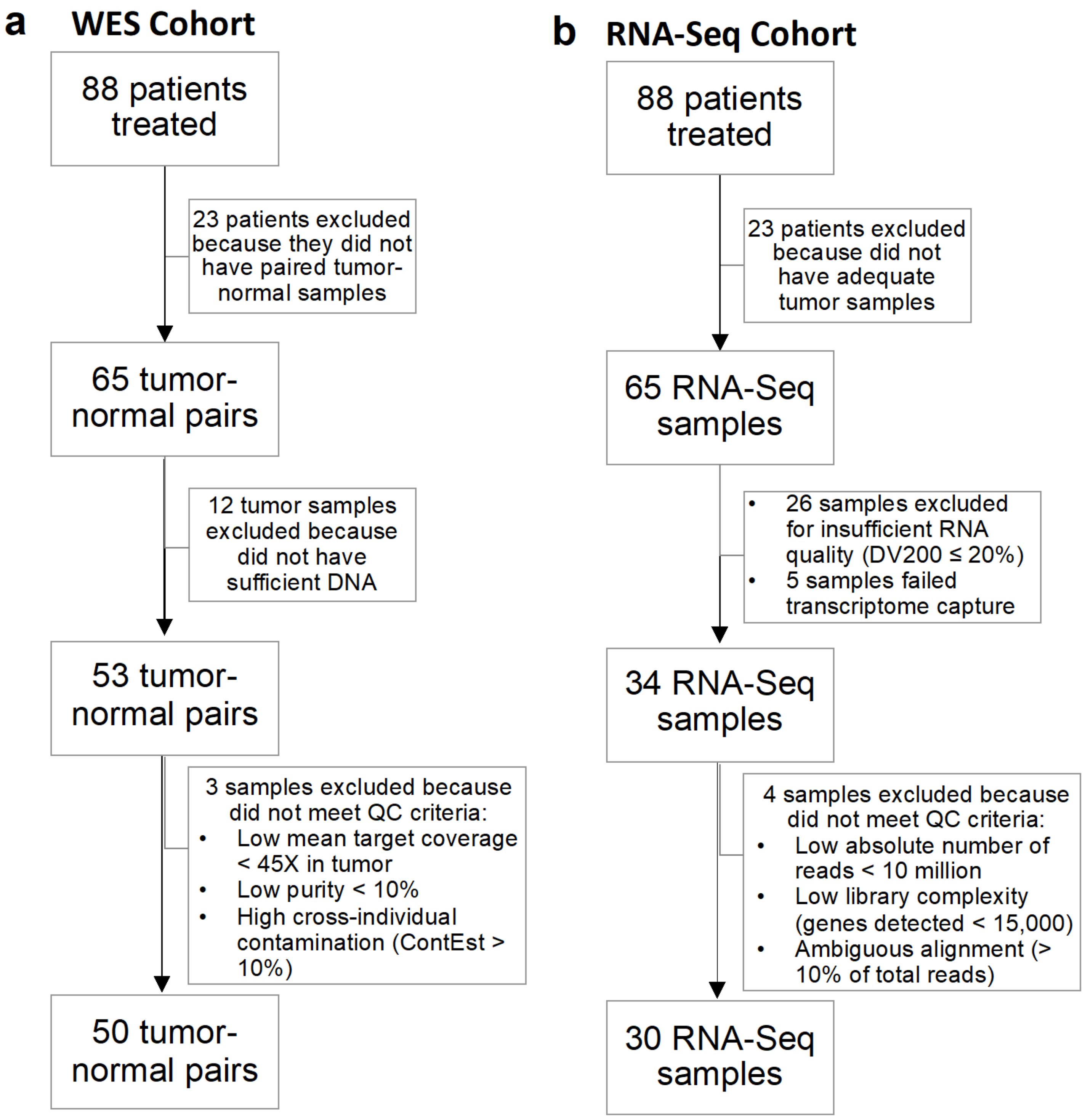

Supplementary Fig. 3: Genomic Cohort Survival and Tumor Mutation Burden

a-d) Genomic cohort survival: Kaplan-Meier curves for PFS in the whole exome sequencing (WES) cohort (**a**) overall and in the (**b**) eribulin pembrolizumab arm stratified by presence vs absence of the APOBEC mutational signature and in the RNA sequencing (RNA-seq) cohort (**c**) overall and in the (**d**) eribulin pembrolizumab arm stratified by PAM50 subtype. Log rank p values are shown. **e-h**) Tumor mutation burden: Nonsynonymous mutational burden in patients with clinical benefit (green) vs. no clinical benefit (yellow) to (**e**) eribulin +/- pembrolizumab overall and (**f**) eribulin alone. Patients with NCB to eribulin alone had marginally higher median TMB than CB patients (**f**), likely due to two high TMB outliers. Nonsynonymous mutational burden by RECIST best response to (**g**) eribulin +/- pembrolizumab overall and (**h**) eribulin alone. Boxplots: box limits indicate the IQR (25th to 75th percentile), with a center line indicating the median. Whiskers show the value ranges up to 1.5 × IQR above the 75th or below the 25th percentile, with outliers beyond those ranges shown as individual points. Unadjusted two-sided Mann-Whitney-Wilcoxon p values are shown.

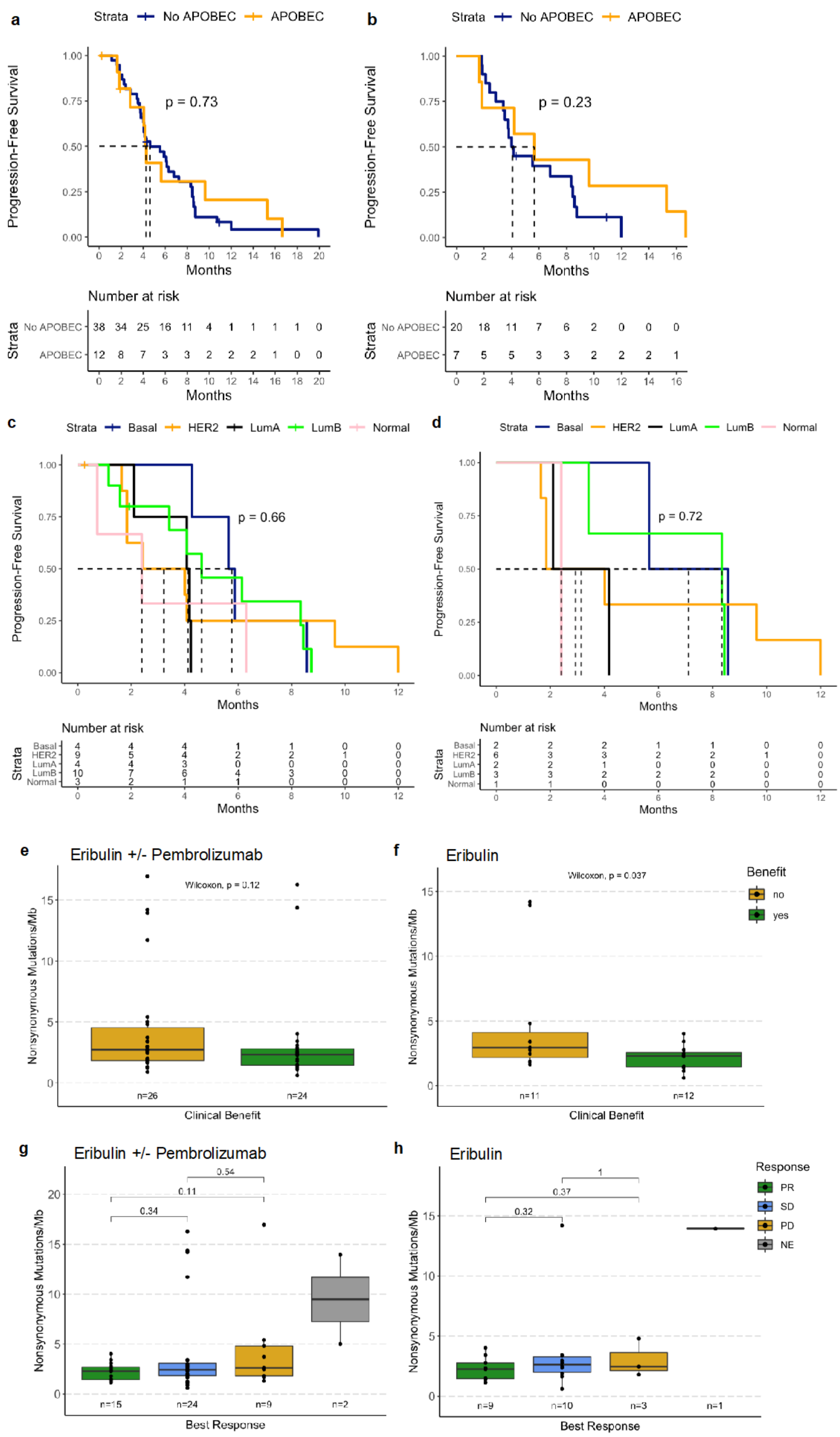

Supplementary Fig. 4: Tumor Ploidy, Heterogeneity, and Purity by Benefit and Response

a-c) Tumor ploidy (**a**), heterogeneity (**b**), and purity (**c**) in patients with clinical benefit (green) vs. no clinical benefit (yellow) and by RECIST best response to eribulin +/- pembrolizumab overall and eribulin alone. Boxplots: box limits indicate the IQR (25th to 75th percentile), with a center line indicating the median. Whiskers show the value ranges up to 1.5 × IQR above the 75th or below the 25th percentile, with outliers beyond those ranges shown as individual points. Unadjusted two-sided Mann-Whitney-Wilcoxon p values are shown. (**d**) Within the eribulin pembrolizumab arm (n = 27), patients with tumors that had top quartile purity, but not top quartile heterogeneity trended, towards having longer progression-free survival. Unadjusted two-sided log rank p values are shown.

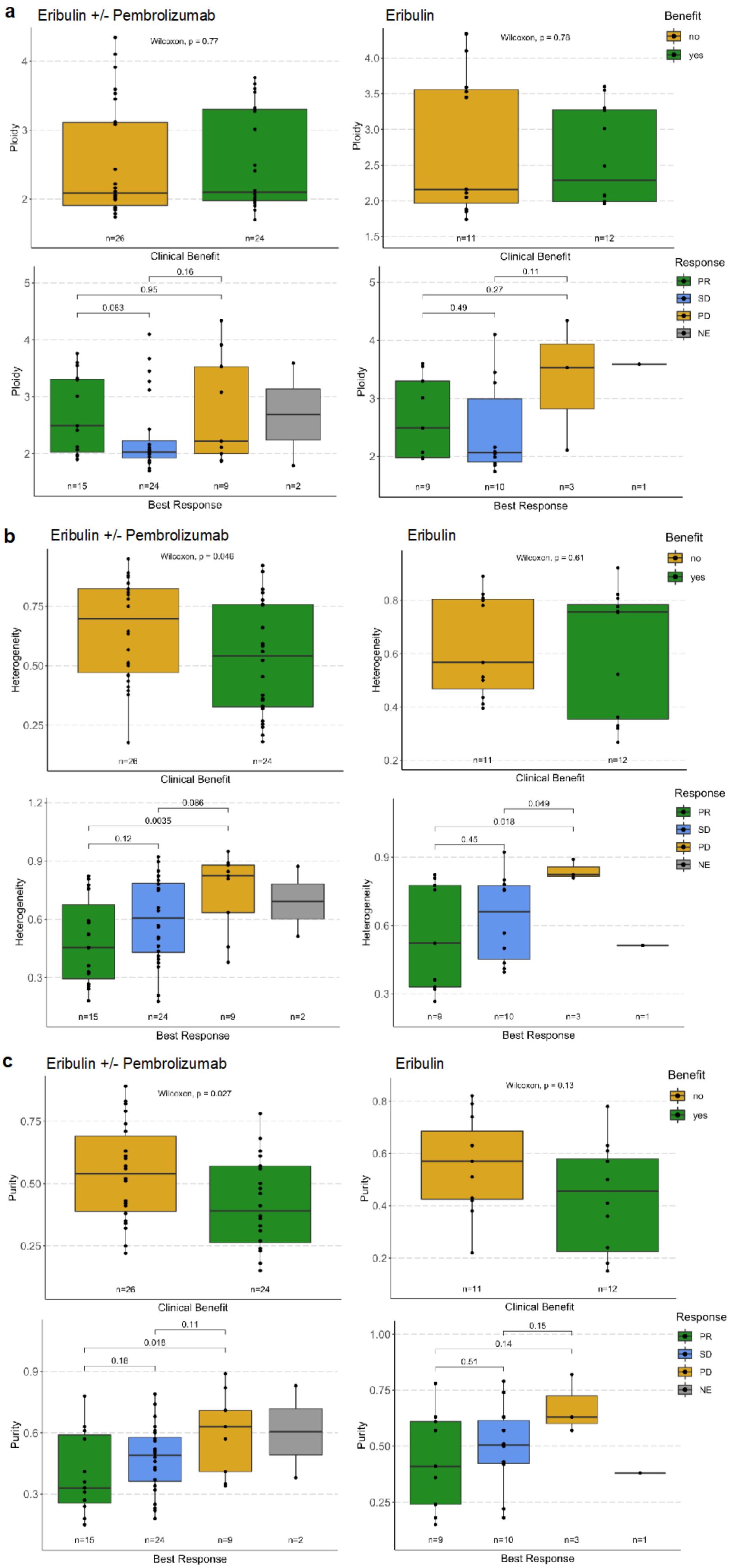

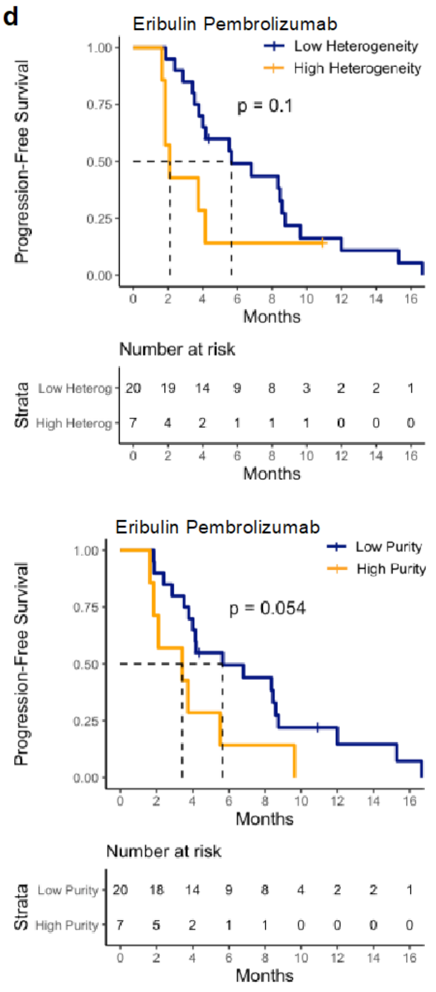

Supplementary Fig. 5: Single-Gene Mutations and Copy Number Alterations

a-f) Single-gene alterations: Single-gene nonsynonymous mutations were not associated with clinical benefit at unadjusted two-sided Fisher's exact $p < 0.05$ prior to multiple hypothesis testing correction in patients treated with (**a**) eribulin and pembrolizumab, (**b**) eribulin +/- pembrolizumab, or (**c**) eribulin alone. Each dot is a gene or group of genes. Amplifications (amp), defined as copy number > 7, and homozygous deletions (del) were not associated with clinical benefit at unadjusted two-sided Fisher's exact $p < 0.04$ prior to multiple hypothesis testing correction in patients treated with (**d**) eribulin and pembrolizumab, (**e**) eribulin +/- pembrolizumab, or (**f**) eribulin alone. Each dot is a gene or group of genes with the size of the dot corresponding to the number of patients. CB, clinical benefit; CNA, copy number alteration; lncRNA, long non-coding RNA; NCB, no clinical benefit.

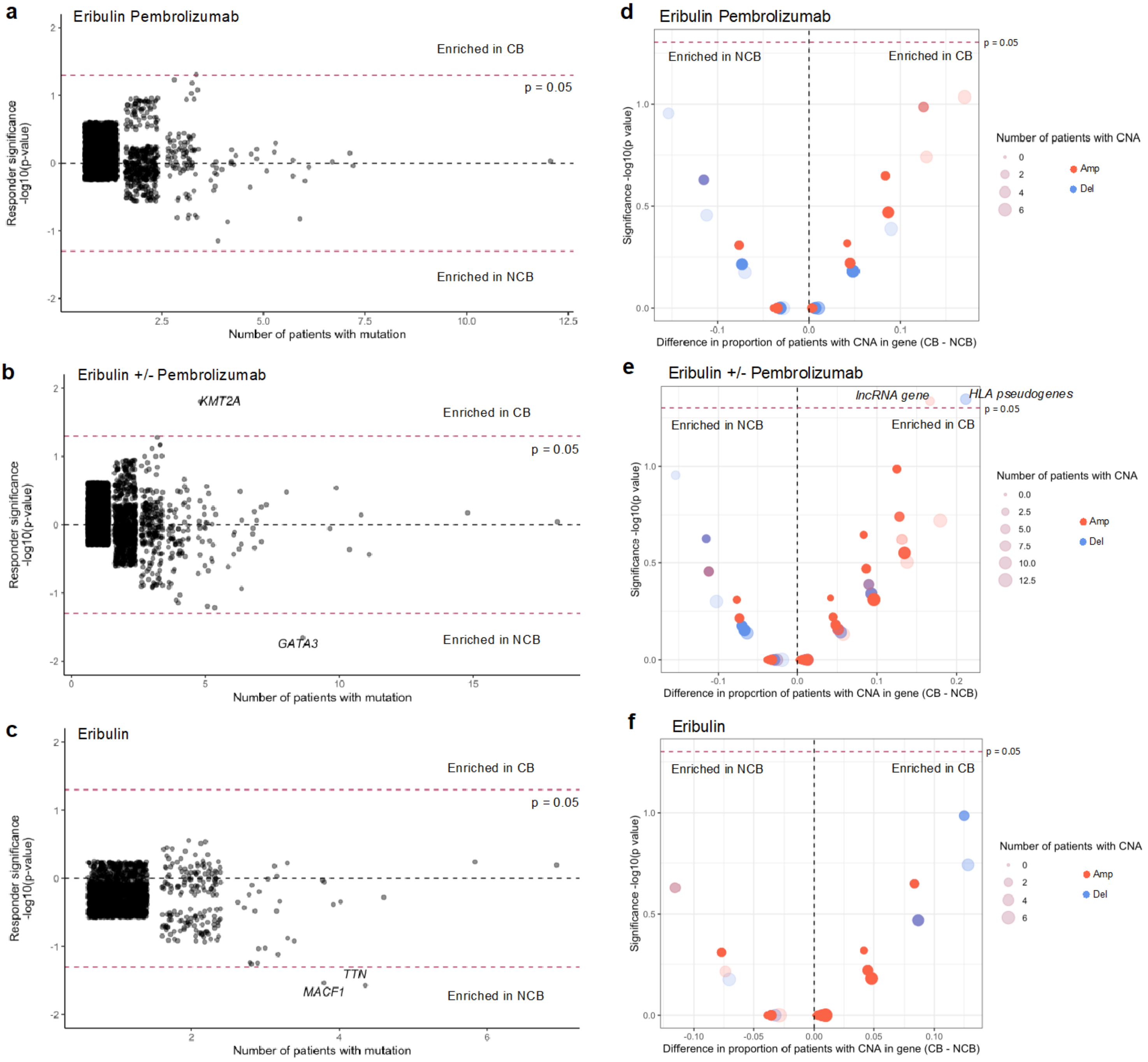

Supplementary Fig. 6: Immune Gene Set Single-Sample Gene Set Enrichment Analysis (ssGSEA)
a-e) ssGSEA of the hallmark immune gene sets[82] showed higher allograft rejection (**a**) and interferon (IFN)-γ (**b**) ssGSEA scores in patients with clinical benefit (green) vs. no clinical benefit (yellow), as well as a trend towards higher inflammatory response (**c**), IL-6/JAK/STAT3 signaling (**d**), and IFN-α (**e**). Boxplots: box limits indicate the IQR (25th to 75th percentile), with a center line indicating the median. Whiskers show the value ranges up to 1.5 × IQR above the 75th or below the 25th percentile, with outliers beyond those ranges shown as individual points. Y axes display ssGSEA scores. Unadjusted two-sided Mann-Whitney-Wilcoxon p values are shown.

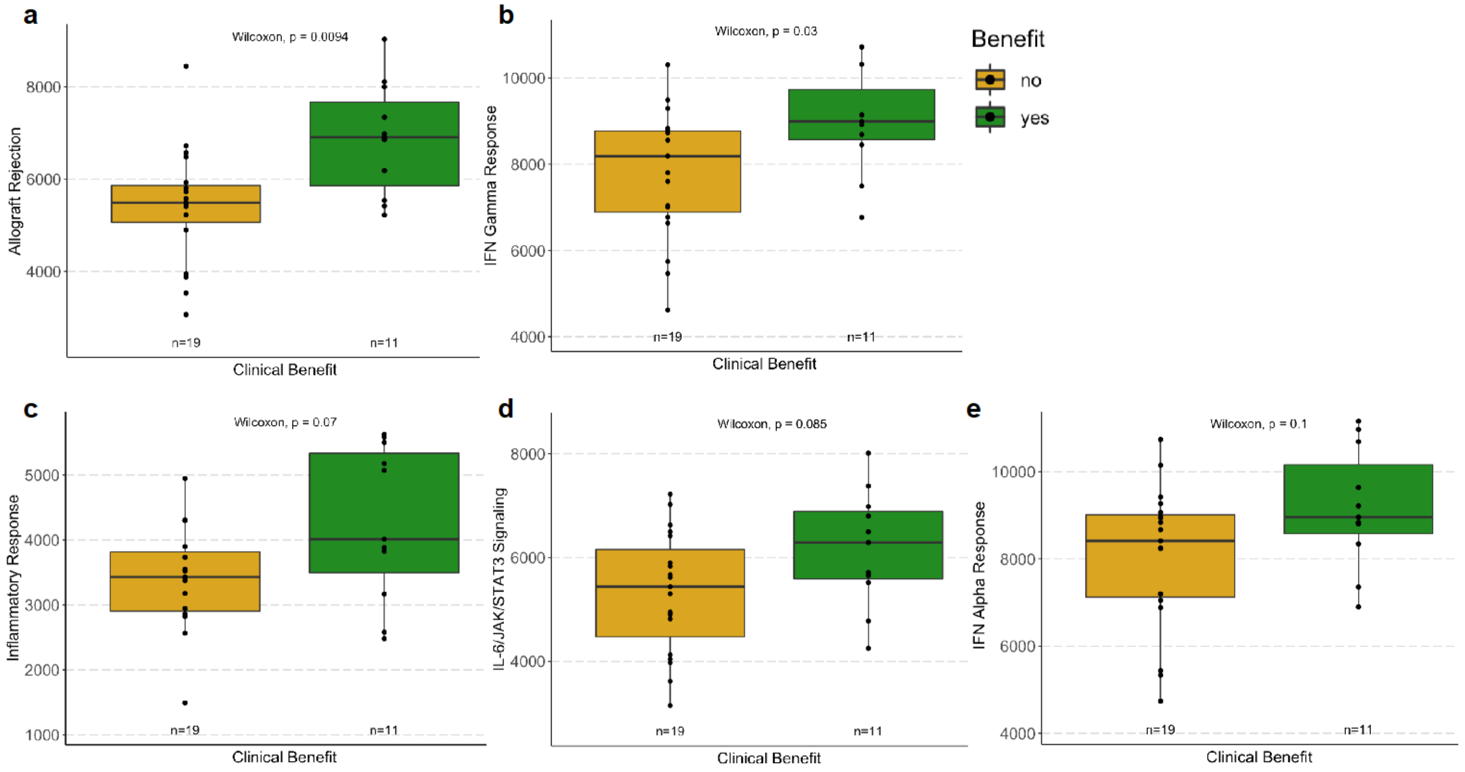

Supplementary Fig. 7: Gene Expression Volcano Plots by Benefit

Volcano plots show the log fold change in expression of allograft rejection pathway (**a-c**) and antigen presentation genes (**d-f**) by clinical benefit (CB) in patients treated with (**a, d**) eribulin and pembrolizumab, (**b, e**) eribulin +/- pembrolizumab, and (**c, f**) eribulin alone. The x axis displays the log2 fold change in expression of the genes between tumors from patients with versus without CB, and the y axis displays the -log10 of the unadjusted two-sided Mann-Whitney-Wilcoxon p value for the gene expression difference in tumors from patients with versus without CB. The allograft rejection pathway is one of the 50 hallmark gene sets,[82] and the antigen presentation gene set is listed in Supplementary Data File 1: Supplementary Table 4. The p values for named genes are listed in Supplementary Data File 1: Supplementary Table 5.

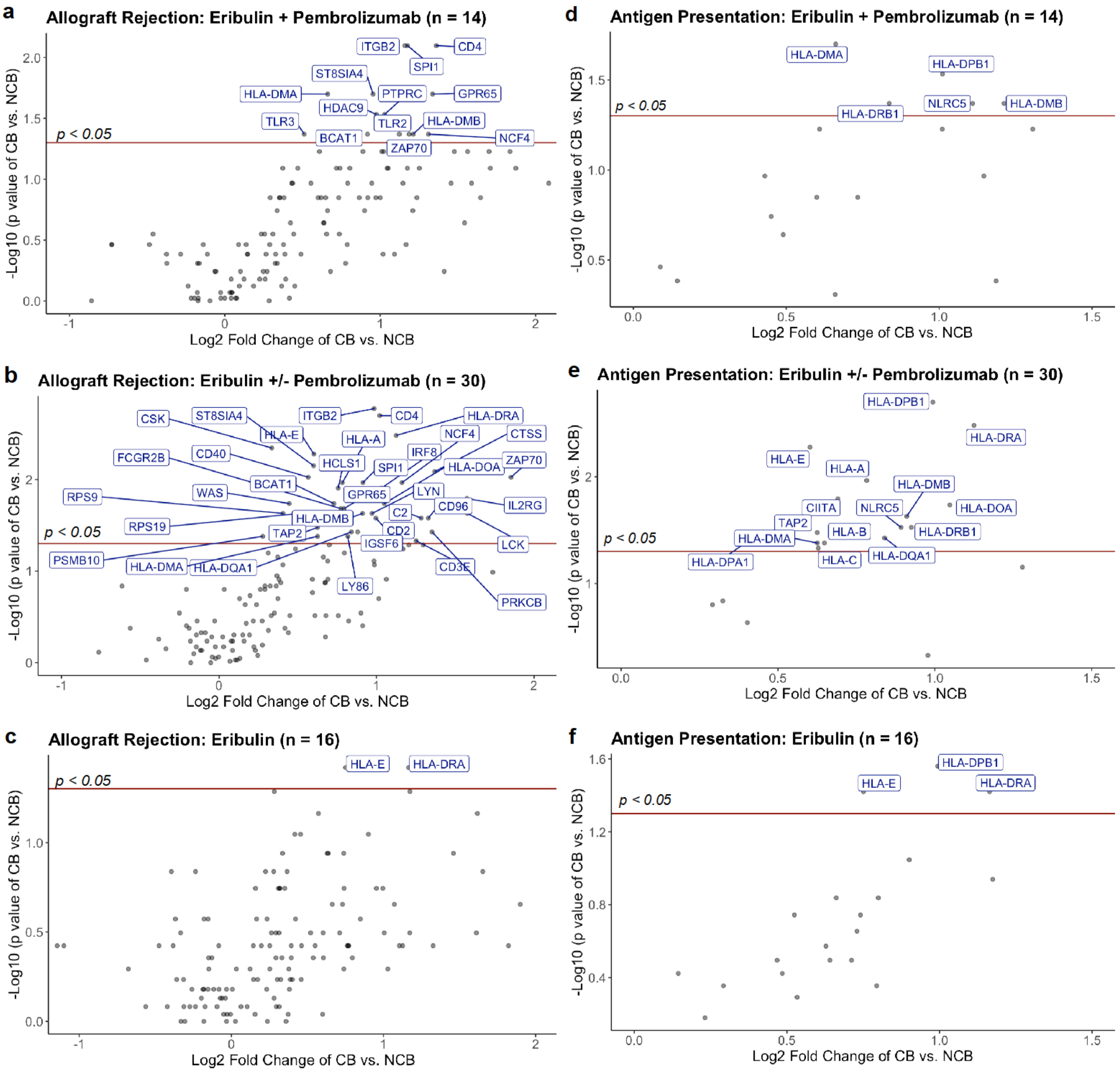

Supplementary Fig. 8: Antigen Presentation Scores and Immune Cell Markers

(**a**) ssGSEA of the antigen presentation gene set (Supplementary Data File 1: Supplementary Table 4) showed higher ssGSEA scores in patients with clinical benefit (green) vs. no clinical benefit (yellow). (**b**, **c**) Kaplan-Meier curves for progression-free survival for patients treated with (**b**) eribulin and pembrolizumab and (**c**) eribulin alone stratified by high versus low antigen presentation (AP) gene set enrichment scores (calculated by ssGSEA and divided by the median) with p values calculated by the log-rank test. (**d**, **e**) The dendritic cell marker gene *ITGAX* had higher gene expression in patients with clinical benefit (green) vs. no clinical benefit (yellow) to eribulin pembrolizumab (**d**) but not to eribulin alone (**e**). Boxplots: box limits indicate the IQR (25th to 75th percentile), with a center line indicating the median. Whiskers show the value ranges up to 1.5 × IQR above the 75th or below the 25th percentile, with outliers beyond those ranges shown as individual points. Y axes display ssGSEA score (**a**) or gene expression in transcripts per million (**d**, **e**). Unadjusted two-sided Mann-Whitney-Wilcoxon p values are shown.

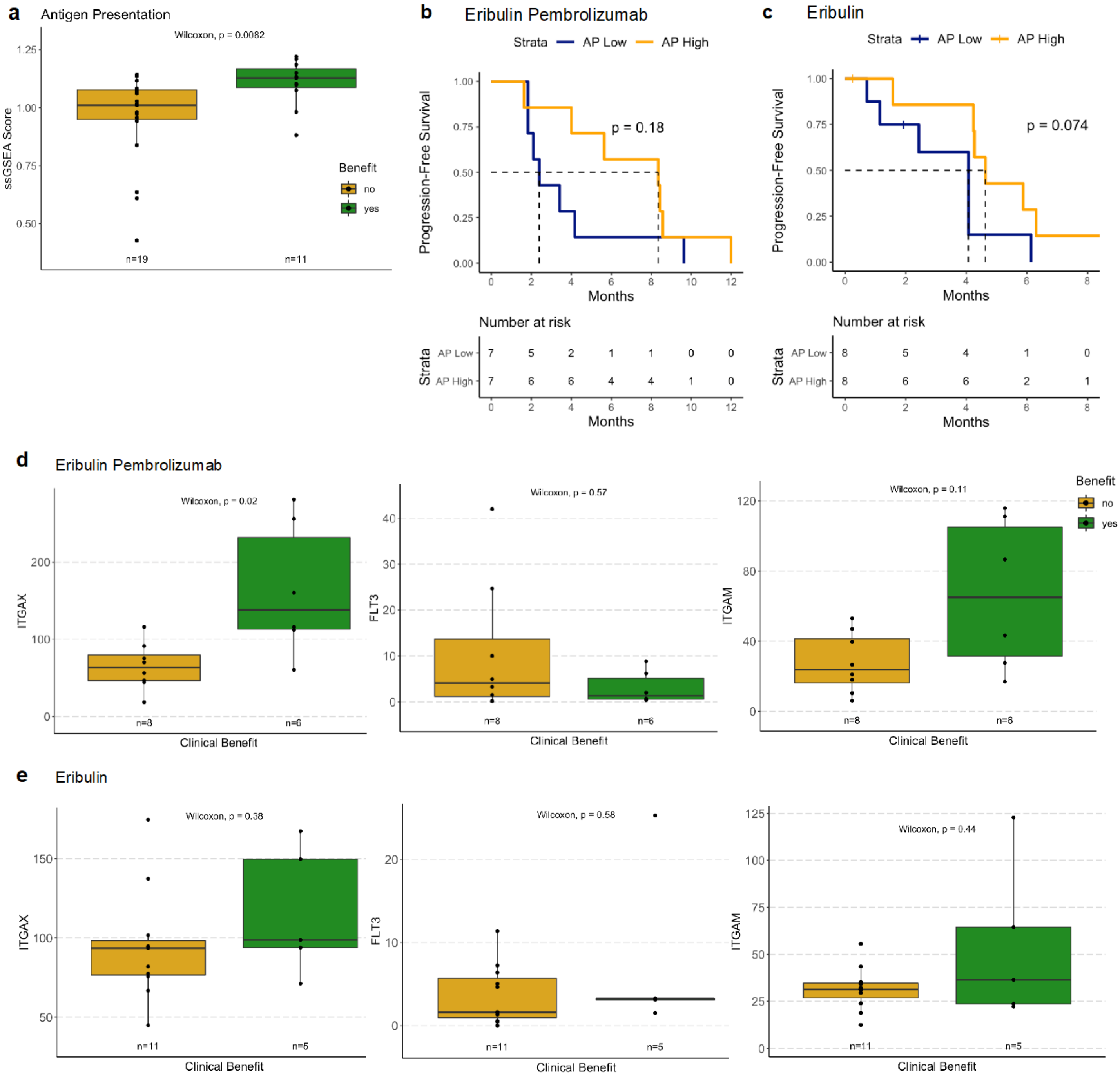

Supplementary Fig. 9: Immune Infiltration and Estrogen Response Gene Expression

a-b) Absolute immune cell infiltrate (**a**) and tumor infiltrating lymphocytes (**b**) in tumors from patients treated with eribulin and pembrolizumab or eribulin alone stratified by clinical benefit (green) versus no clinical benefit (yellow). **c-d**) ssGSEA of the hallmark estrogen gene sets[82] showed a trend towards higher early (**c**) and late (**d**) estrogen response ssGSEA scores in patients with no clinical benefit (yellow) vs. clinical benefit (green). Boxplots: box limits indicate the IQR (25th to 75th percentile), with a center line indicating the median. Whiskers show the value ranges up to 1.5 × IQR above the 75th or below the 25th percentile, with outliers beyond those ranges shown as individual points. Y axes display ssGSEA scores. Unadjusted two-sided Mann-Whitney-Wilcoxon p values are shown. **e-f**) Volcano plots show the log fold change in expression of antigen presentation genes (Supplementary Data File 1: Supplementary Table 4) by early (**e**) and late (**f**) estrogen response pathway expression. The x axis displays the log2 fold change in expression of the genes between tumors in the lowest vs. highest tertile of hallmark early or late estrogen response ssGSEA scores. The y axis displays the -log10 of the unadjusted two-sided Mann-Whitney-Wilcoxon p value for the gene expression difference in tumors in the lowest vs. highest tertile of early or late estrogen response ssGSEA scores. The p values are listed in Supplementary Data File 1: Supplementary Table 6.

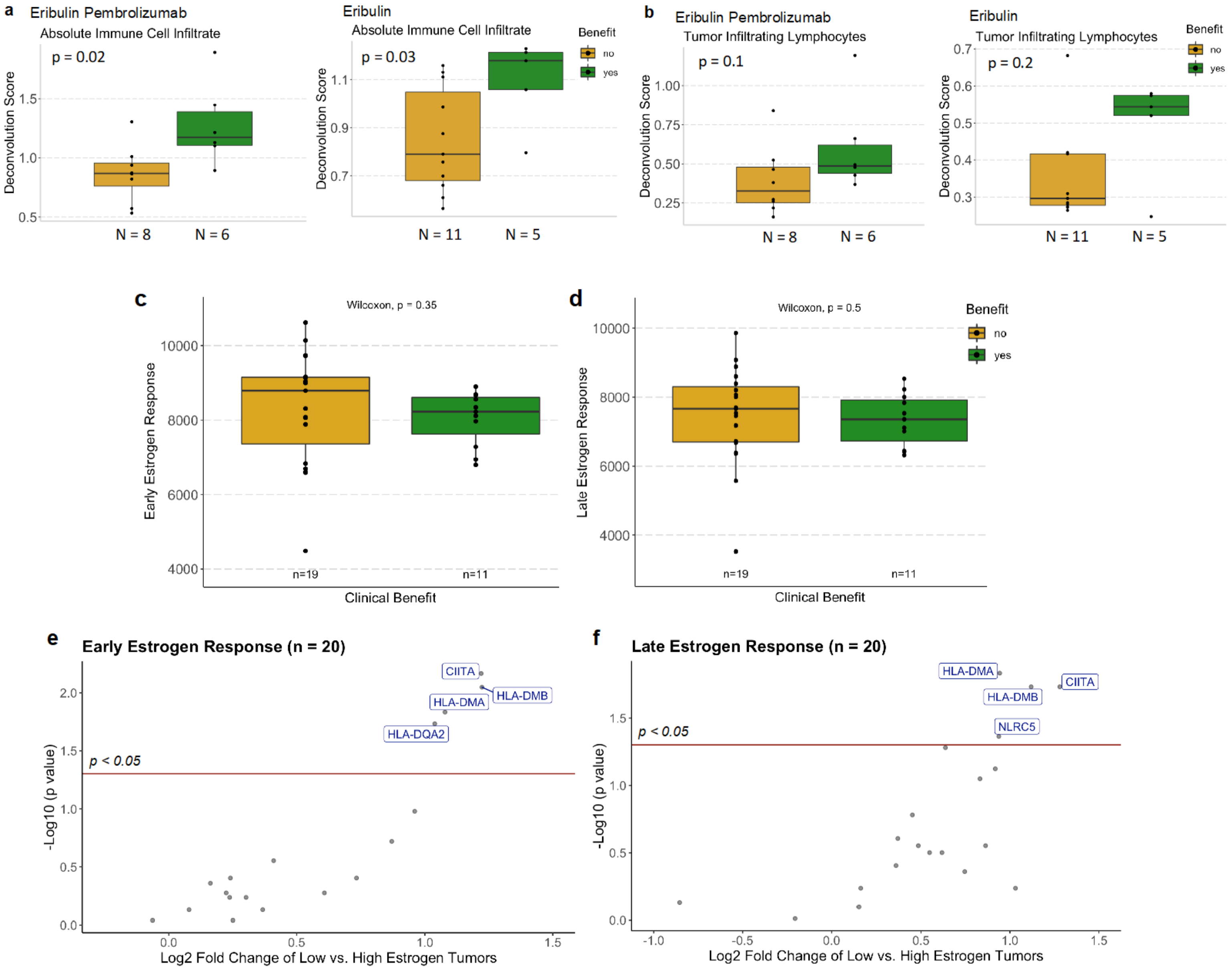

Supplementary Fig. 10: Cytokines by Response and Toxicity

(**a**) Pre- and on-treatment levels of MHC class I polypeptide-related sequence B (MICB) stratified by clinical benefit (green) versus no clinical benefit (yellow) with two-sided Van Elteren p values adjusted for treatment group. (**b-g**) Pre- and on-treatment levels of macrophage inflammatory protein 1-alpha (MIP1a) (**b**), MICB (**c**), interleukin-2 receptor alpha chain (IL-2RA) (**d**), interleukin-23 (IL-23) (**e**), angiopoietin-2 (Ang2) (**f**), and fractalkine (**g**) stratified by treatment toxicity group with unadjusted two-sided Mann-Whitney-Wilcoxon p values. Boxplots: box limits indicate the IQR (25th to 75th percentile), with a center line indicating the median. Whiskers show the value ranges up to 1.5 × IQR above the 75th or below the 25th percentile, with outliers beyond those ranges shown as individual points. E, eribulin; EP, eribulin pembrolizumab; irAE, immune-related adverse event.

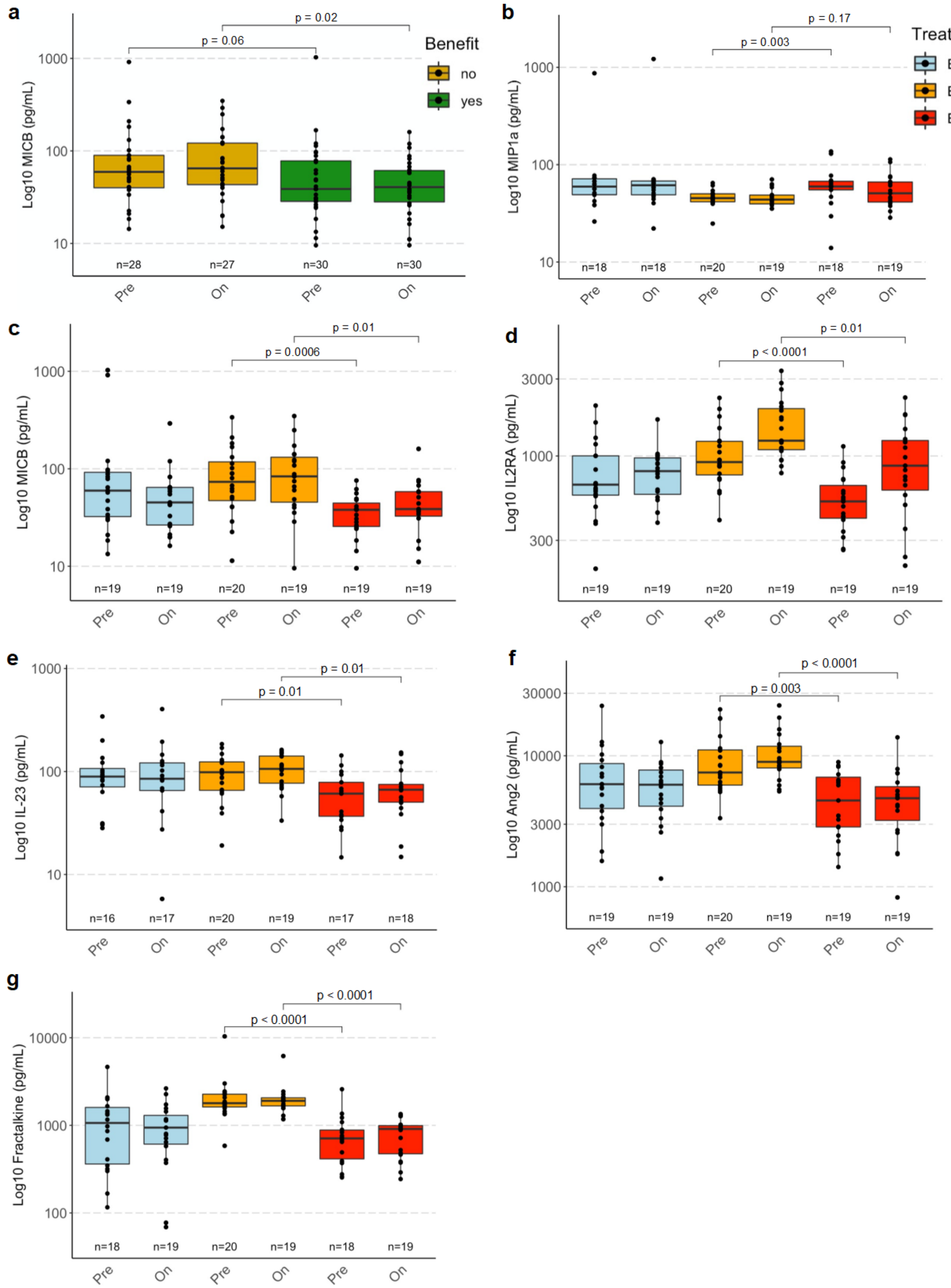

Supplement: Supplementary file 1 — Supplementary Information [file 41467_2021_25769_MOESM1_ESM.pdf]
