# Supplementary material for: Molecular correlates of response to eribulin and pembrolizumab in hormone receptor-positive metastatic breast cancer
Source: Nat Commun. 2021 Sep 21;12:5563. doi: 10.1038/s41467-021-25769-z (PMC8455578; doi:10.1038/s41467-021-25769-z)
Supplement: Supplementary file 2 — Description of Additional Supplementary Files [file 41467_2021_25769_MOESM2_ESM.docx]

Description of Additional Supplementary File

Title: Supplementary Data 1

Description: This file contains supplementary tables referenced in the manuscript.
